# C2-Alkylation of *N*-pyrimidylindole with vinylsilane via cobalt-catalyzed C–H bond activation

**DOI:** 10.3762/bjoc.8.174

**Published:** 2012-09-14

**Authors:** Zhenhua Ding, Naohiko Yoshikai

**Affiliations:** 1 Division of Chemistry and Biological Chemistry, School of Physical and Mathematical Sciences, Nanyang Technological University, Singapore 637371, Singapore

**Keywords:** alkylation, C–H functionalization, cobalt, indole, vinylsilane

## Abstract

Direct C2-alkylation of an indole bearing a readily removable *N*-pyrimidyl group with a vinylsilane was achieved by using a cobalt catalyst generated in situ from CoBr_2_, bathocuproine, and cyclohexylmagnesium bromide. The reaction allows coupling between a series of *N*-pyrimidylindoles and vinylsilanes at a mild reaction temperature of 60 °C, affording the corresponding alkylated indoles in moderate to good yields.

## Introduction

The indole ring ubiquitously occurs in biologically active natural and unnatural compounds [1–3]. Consequently, there has been a strong demand for catalytic methods allowing efficient and regioselective functionalization of indole derivatives [4–6]. Over the past decade, transition-metal-catalyzed direct functionalization has emerged as a powerful strategy for the direct introduction of aryl and alkenyl groups to the C2 and C3 positions of indole [7–9]. The situation is different when it comes to direct C–H alkylation [10–11]. The intrinsically nucleophilic C3 position of indole is amenable to a variety of catalytic alkylation reactions such as Friedel–Crafts reaction [5]. On the other hand, C2-alkylation of indoles has traditionally required 2-lithioindoles generated by C2-lithiation with a stoichiometric lithium base or indol-2-yl radicals generated from 2-halogenated indoles [12–17]. Examples of direct C2-alkylation via transition-metal-catalyzed C–H activation are still limited [18–20], while Jiao and Bach recently reported an elegant palladium-catalyzed, norbornene-mediated C2-alkylation reaction with a broad spectrum of alkyl bromides [21].

**Scheme 1:**
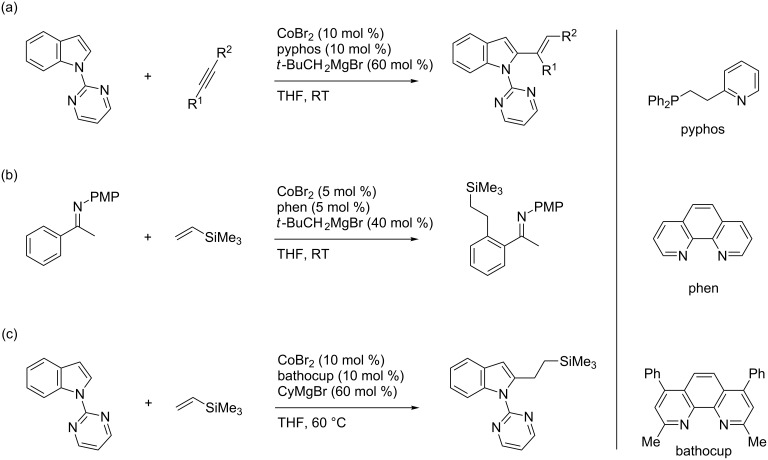
(a) Cobalt-catalyzed C2-alkenylation of *N*-pyrimidylindole, (b) ortho-alkylation of aryl imine, and (c) C2-alkylation of *N*-pyrimidylindole.

Over the past few years, our group and others have explored C–H bond functionalization reactions using cobalt complexes as inexpensive transition-metal catalysts [22], which often feature mild reaction conditions and unique regioselectivities [23–32]. As a part of this research program, we have recently reported a C2-alkenylation reaction of *N*-pyrimidylindoles with internal alkynes catalyzed by a cobalt–pyridylphosphine complex (Scheme 1a) [33], in which the pyrimidyl group functions as a readily removable directing group [34]. We also reported an ortho-alkylation reaction of aromatic imines with vinylsilanes and simple olefins using a cobalt–phenanthroline catalyst (Scheme 1b) [35]. Building on these studies, we have developed a cobalt–bathocuproine catalyst for the direct C2-alkylation reaction of *N*-pyrimidylindoles with vinylsilanes, which is reported herein (Scheme 1c).

## Results and Discussion

Our study commenced with the optimization of the reaction of *N*-pyrimidylindole **1a** with vinyltrimethylsilane (**2a**). The combination of CoBr_2_ (10 mol %), 1,10-phenanthroline (phen, 10 mol %) and neopentylmagnesium bromide (100 mol %), which was effective for ortho-alkylation of aromatic imines [35], afforded the desired adduct **3aa** in only 17% yield accompanied by a small amount of a C2-neopentylated product **4** (Table 1, entry 1). Subsequent examination of phenanthroline and bipyridine-type ligands (Table 1, entries 2–5) revealed that 2,9-dimethyl-1,10-phenanthroline (neocuproine) and 2,9-dimethyl-4,7-diphenylphenanthroline (bathocuproine) improved the yield of **3aa**, while the byproduct **4** could not be suppressed (Table 1, entries 3 and 4). The *P*,*N*-bidentate ligand pyphos, which was the optimum ligand for the alkenylation reaction [33], was poorly effective (Table 1, entry 6). Additional screening of *N*-heterocyclic carbene (NHC) and phosphine ligands did not lead to an improvement of the catalytic efficiency (Table 1, entries 7–9). The reaction turned out to be sensitive to the amount of the Grignard reagent, as reduction of its loading from 100 to 60 mol % improved the yield of **3aa** while suppressing the formation of byproduct **4** (Table 1, entry 10).

**Table 1:** Screening of ligands.^a^

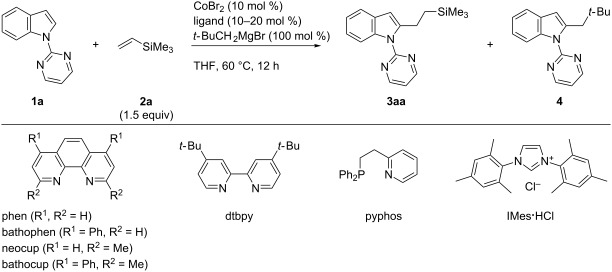			

entry	ligand (mol %)	yield (%)^b^	
		**3aa**	**4**

1	phen (10)	17	7
2	bathophen (10)	11	7
3	neocup (10)	32	12
4	bathocup (10)	34	20
5	dtbpy (10)	1	3
6	pyphos (10)	2	3
7	IMes·HCl (10)	4	2
8	PPh_3_ (20)	9	5
9	P(3-ClC_6_H_4_)_3_ (20)	23	11
10^c^	bathocup	50	10

^a^Reaction was performed on a 0.3 mmol scale. ^b^Determined by GC using *n*-tridecane as an internal standard. ^c^60 mol % of *t-*BuCH_2_MgBr was used.

Next, we performed screening of Grignard reagents using bathocuproine as the ligand (Table 2). Among Grignard reagents without β-hydrogen atoms, neopentyl- and phenylmagnesium bromides afforded **3aa** in comparable yields (Table 2, entries 1 and 4), while trimethylsilylmethyl- and methylmagnesium chlorides gave much poorer results (Table 2, entries 2 and 3). Primary and secondary alkyl Grignard reagents also promoted the reaction, in which the reaction efficiency was strongly dependent on the alkyl group (Table 2, entries 5–10). We identified cyclohexylmagnesium bromide as the optimum Grignard reagent, which afforded **3aa** in 69% isolated yield without formation of the cross-coupling product **4** between **1a** and the Grignard reagent.

**Table 2:** Screening of Grignard reagents.^a^

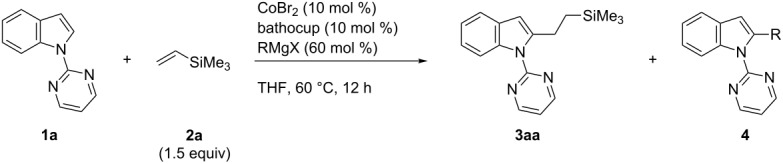			

entry	RMgX	yield (%)^b^	
		**3aa**	**4**

1	*t-*BuCH_2_MgBr	50	10
2	Me_3_SiCH_2_MgCl	26	5
3	MeMgCl	14	4
4	PhMgBr	46	5
5	EtMgBr	28	3
6	BuMgBr	45	0
7	*i*-PrMgBr	49	3
8	*c*-C_3_H_5_MgBr	13	0
9	*c*-C_5_H_9_MgBr	46	0
10	*c*-C_6_H_11_MgBr	67 (69)^c^	0

^a^Reaction was performed on a 0.3 mmol scale. ^b^Determined by GC using *n*-tridecane as an internal standard. ^c^Isolated yield.

**Scheme 2:**
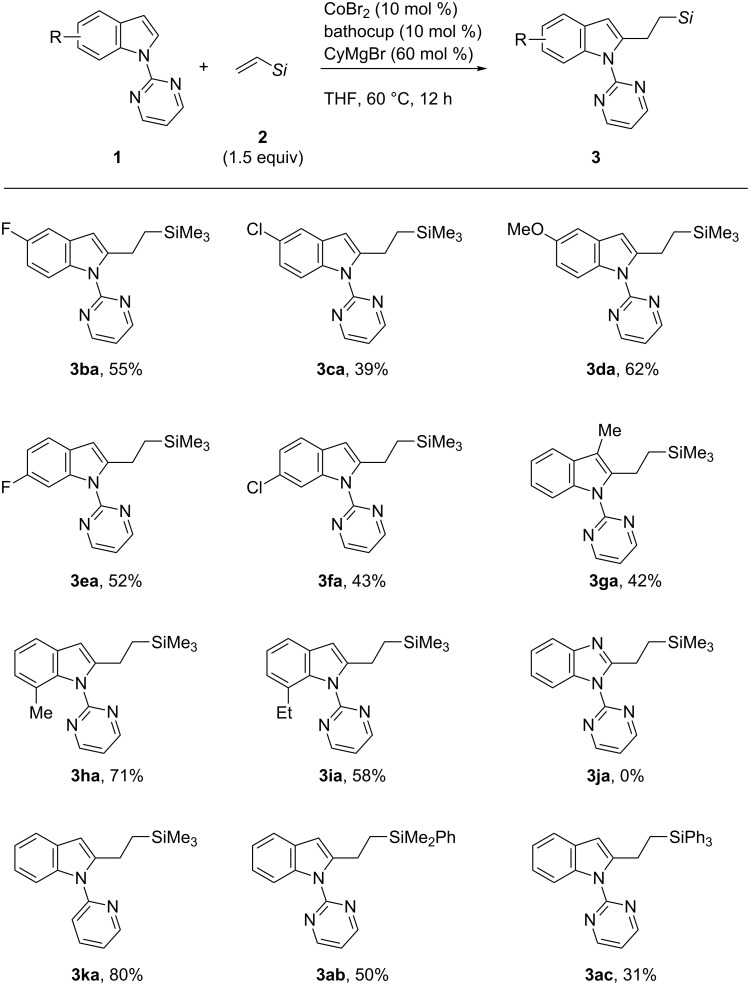
Addition of *N*-pyrimidylindoles to vinylsilanes.

With the optimized catalytic system in hand, we explored the scope of the reaction (Scheme 2). A variety of *N*-pyrimidylindoles participated in the reaction with vinyltrimethylsilane to afford the alkylation products **3ba**–**3ia** in moderate yields, with tolerance of electron-withdrawing (F and Cl) and electron-donating (OMe) substituents and steric hindrance at the C3 and C7 positions. Unlike the cobalt-catalyzed C2-alkenylation reaction (Scheme 1a) [33], the reaction did not tolerate a cyano group on the indole substrate. In addition, *N*-pyrimidyl benzimidazole did not participate in the present alkylation reaction, although it was a good substrate for the C2-alkenylation reaction. A pyridyl group served as an alternative directing group to the pyrimidyl group, affording the alkylation product **3ka** in 80% yield. On the other hand, an *N*,*N*-dimethylcarbamoyl group, which was previously used as a directing group for rhodium-catalyzed C2-alkenylation [36], was entirely ineffective. Vinylsilanes bearing dimethylphenylsilyl and triphenylsilyl groups were amenable to the addition reaction with **1a**, affording the adduct **3ab** and **3ac** in modest yields. Vinyltriethoxysilane also reacted with **1a** in 20% yield, although the product could not be separated in a pure form.

**Scheme 3:**
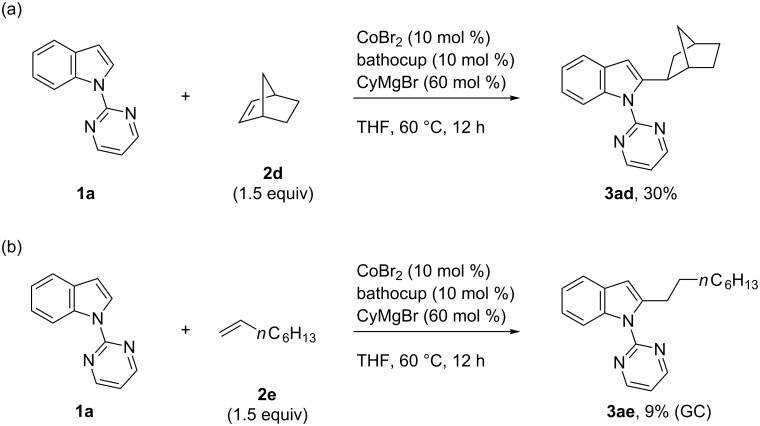
Addition of *N*-pyrimidylindole to norbornene (a) and 1-octene (b).

Unfortunately, the present catalytic system was not very effective for C2-alkylation with simple olefins. The reaction of **1a** with norbornene (**2d**) afforded the alkylation product **3ad** in 30% yield (Scheme 3a). The reaction of 1-octene (**2e**) was even more sluggish, affording the alkylation product **3ae** in only 9% yield (Scheme 3b). Styrene also reacted rather sluggishly to afford only a small amount of the alkylation product (3% as estimated by GC and GCMS), the regiochemistry (branched versus linear) of which has yet to be determined. An acrylate ester was not tolerable as an olefinic reaction partner because of the presence of excess Grignard reagent.

**Scheme 4:**
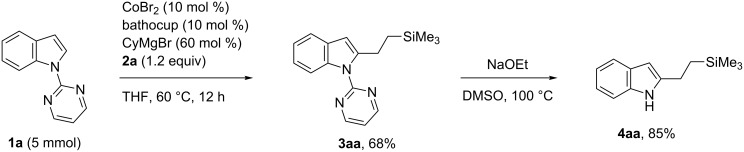
Gram-scale reaction and deprotection of *N*-pyrimidyl group.

The present alkylation reaction could be performed on a preparatively useful scale. Thus, alkylation of **1a** with vinyltrimethylsilane (**2a**) on a 5 mmol scale afforded the adduct **3aa** in 68% yield (Scheme 4). Furthermore, the pyrimidyl group on **3aa** could be readily removed by heating with NaOEt in DMSO, affording the free indole **4aa** in 85% yield.

## Conclusion

In summary, we have developed a cobalt–bathocuproine catalyst for C2-alkylation of *N*-pyrimidyl indoles with vinylsilanes. The reaction could be performed at a mild temperature of 60 °C, on a preparatively useful scale. Ensuing studies will focus on the development of more broadly applicable catalytic systems for the direct alkylation of indole and other heterocycles.

## Experimental

### 

#### Typical procedure: Cobalt-catalyzed alkylation of *N*-pyrimidyl indole **1a** with vinylsilane **2a**

In a Schlenk tube were placed 1-(pyrimidin-2-yl)-1*H*-indole (**1a**) (58.6 mg, 0.3 mmol), CoBr_2_ (6.6 mg, 0.03 mmol), and bathocuproine (10.8 mg, 0.03 mmol), which were then dissolved in THF (1.3 mL). To the solution was added cyclohexylmagnesium bromide (0.60 M in THF, 0.3 mL, 0.18 mmol) at 0 °C. After stirring for 30 min at this temperature, vinyltrimethylsilane (**2a**) (66 μL, 0.45 mmol) was added. The reaction mixture was stirred at 60 °C for 12 h, and then quenched with saturated aqueous solution of NH_4_Cl (1.5 mL). The resulting mixture was extracted with ethyl acetate (3 × 10 mL). The combined organic layer was dried over Na_2_SO_4_ and concentrated under reduced pressure. Purification of the crude product by silica gel chromatography (eluent: hexane/EtOAc 100:1) afforded the title compound as a colorless oil (61.2 mg, 69%).

## Supporting Information

File 1 Experimental details and characterization data of new compounds.
