# Supplementary material for: C2-Alkylation of N-pyrimidylindole with vinylsilane via cobalt-catalyzed C–H bond activation
Source: Beilstein J Org Chem. 2012 Sep 14;8:1536–42. doi: 10.3762/bjoc.8.174 (PMC3458779; doi:10.3762/bjoc.8.174)
Supplement: File 1 — Experimental details and characterization data of new compounds. [file Beilstein_J_Org_Chem-08-1536-s001.pdf]

Supporting Information

for

C2-Alkylation of *N*-pyrimidylindole with vinylsilane via cobalt-catalyzed C–H bond activation

Zhenhua Ding and Naohiko Yoshikai*

Address: Division of Chemistry and Biological Chemistry, School of Physical and Mathematical Sciences, Nanyang Technological University, Singapore 637371, Singapore

Email: Naohiko Yoshikai - nyoshikai@ntu.edu.sg

* Corresponding author

Experimental details and characterization data of new compounds

Contents

Materials and Methods S2

C2-Alkylation of *N*-pyrimidyl indole with vinylsilane S3

Removal of the pyrimidyl group S9

References S9

^{1}H and ^{13}C NMR spectra S10

Materials and Methods

General. All reactions dealing with air or moisture-sensitive compounds were performed by standard Schlenk techniques in oven-dried reaction vessels under a nitrogen atmosphere. Analytical thin-layer chromatography (TLC) was performed on Merck 60 F254 silica gel plates. ^{1}H and ^{13}C nuclear magnetic resonance (NMR) spectra were recorded on a Bruker AV-400 (400 MHz/100 MHz) NMR spectrometer. ^{1}H and ^{13}C NMR spectra are reported in parts per million (ppm) downfield from an internal standard, tetramethylsilane (0 ppm) and $CHCl_3$ (77.0 ppm), respectively. Gas chromatographic (GC) analysis was performed on a Shimadzu GC-2010 system equipped with an FID detector and a capillary column, DB-5 (Agilent J&W, 0.25 mm i.d. x 30 m, 0.25 μm film thickness). High-resolution mass spectra (HRMS) were obtained with a Q-Tof Premier LC HR mass spectrometer.

Materials. Unless otherwise noted, reagents were purchased from Aldrich, Alfa Aesar, and other commercial suppliers and were used as received. THF was distilled over Na/benzophenone. Cyclohexylmagnesium bromide was prepared from bromocyclohexane and magnesium turnings in anhydrous THF and titrated before use. Sodium ethoxide was prepared from sodium and ethanol just before use. Anhydrous $CoBr_2$ (99%) was purchased from Aldrich. *N*-Pyrimidyl indoles were prepared according to the literature procedure [1,2].

C2-Alkylation of *N*-pyrimidyl indole with vinylsilane

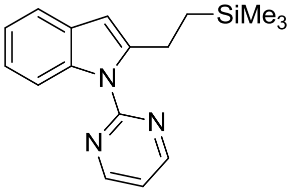

Typical Procedure: 1-(Pyrimidin-2-yl)-2-(2-(trimethylsilyl)ethyl)-1*H*-indole (3aa). In a Schlenk tube were placed 1-(pyrimidin-2-yl)-1*H*-indole (58.6 mg, 0.3 mmol), $CoBr_2$ (6.6 mg, 0.03 mmol), and bathocuproine (10.8 mg, 0.03 mmol), which were then dissolved in THF (1.3 mL). To the solution was added cyclohexylmagnesium bromide (0.60 M in THF, 0.3 mL, 0.18 mmol) at 0 °C. After stirring for 30 min at this temperature, vinyltrimethylsilane (66 μL, 0.45 mmol) was added. The reaction mixture was stirred at 60 °C for 12 h, and then quenched with saturated aqueous solution of NH_4Cl (1.5 mL). The resulting mixture was extracted with ethyl acetate (10 mL × 3). The combined organic layer was dried over Na_2SO_4 and concentrated under reduced pressure. Purification of the crude product by silica gel chromatography (eluent: hexane/EtOAc = 100/1) afforded the title compound as a colorless oil (61.2 mg, 69%). The 5 mmol scale reaction was performed according to a similar procedure by using a 100 mL Schlenk tube to afford the same product in 68% yield.

R_f 0.39 (hexane/EtOAc = 10/1); ^{1}H NMR (400 MHz, $CDCl_3$): δ 8.80 (d, *J* = 4.8 Hz, 2 H), 8.24 (d, *J* = 8.0 Hz, 1 H), 7.54–7.52 (m, 1 H), 7.23–7.13 (m, 3 H), 6.50 (s, 1 H), 3.20–3.16 (m, 2 H), 0.86–0.82 (m, 2 H), 0.02 (s, 9 H); ^{13}C NMR (100 MHz, $CDCl_3$): δ 158.3, 158.1, 145.0, 137.1, 129.4, 122.3, 121.7, 119.6, 117.0, 113.7, 105.1, 23.8, 16.5, −1.8; HRMS (ESI) Calcd for $C_{17}H_{21}N_3Si$ $[M + H]^+$ 296.1583, found 296.1584.

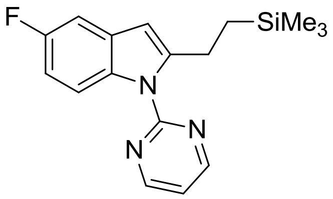

5-Fluoro-1-(pyrimidin-2-yl)-2-(2-(trimethylsilyl)ethyl)-1*H*-indole (3ba): 55% yield; colorless oil; R_f 0.37 (hexane/EtOAc = 10/1); ^{1}H NMR (400 MHz, $CDCl_3$): δ 8.78 (d, *J* = 4.8 Hz, 2 H), 8.23–8.19 (m, 1 H), 7.18–7.14 (m, 2 H), 6.95–6.90 (m, 1 H), 6.45 (s, 1 H), 3.19–3.15 (m,

2 H), 0.85–0.81 (m, 2 H), 0.02 (s, 9 H); ^{13}C NMR (100 MHz, CDCl$_3$): δ 158.9 (d, $^1J_{C-F}$ = 236 Hz), 158.2, 158.1, 146.8, 133.4, 130.1 (d, $^3J_{C-F}$ = 10.0 Hz), 117.1, 114.8 (d, $^3J_{C-F}$ = 9.1 Hz), 109.9 (d, $^2J_{C-F}$ = 24.8 Hz), 104.9, 104.8 (d, $^2J_{C-F}$ = 27.0 Hz), 24.1, 16.5, -1.8; HRMS (ESI) Calcd for $C_{17}H_{20}FN_3Si$ [M + H]$^+$ 314.1489, found 314.1491.

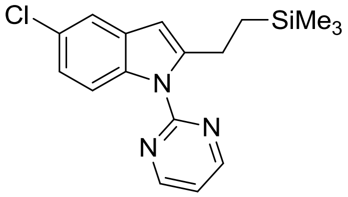

5-Chloro-1-(pyrimidin-2-yl)-2-(2-(trimethylsilyl)ethyl)-1*H*-indole (3ca): 39% yield; yellow oil; R_f 0.38 (hexane/EtOAc = 10/1); ^{1}H NMR (400 MHz, CDCl$_3$): δ 8.79 (d, *J* = 4.8 Hz, 2 H), 8.17 (d, *J* = 8.8 Hz, 1 H), 7.47 (d, *J* = 2.1 Hz, 1 H), 7.19–7.13 (m, 2 H), 6.43 (s, 1 H), 3.18–3.14 (m, 2 H), 0.84–0.80 (m, 2 H), 0.01 (s, 9 H); ^{13}C NMR (100 MHz, CDCl$_3$): δ 158.14, 158.09, 146.5, 135.4, 130.5, 127.1, 122.3, 119.1, 117.2, 114.9, 104.5, 24.0, 16.4, −1.8; HRMS (ESI) Calcd for $C_{17}H_{20}ClN_3Si$ [M + H]$^+$ 330.1193, found 330.1190.

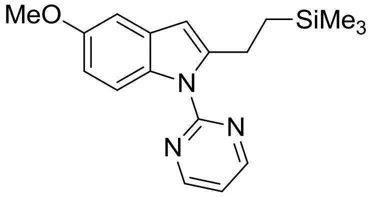

5-Methoxy-1-(pyrimidin-2-yl)-2-(2-(trimethylsilyl)ethyl)-1*H*-indole (3da): 62% yield; colorless oil; R_f 0.32 (hexane/EtOAc = 10/1); ^{1}H NMR (400 MHz, CDCl$_3$): δ 8.75 (d, *J* = 4.8 Hz, 2 H), 8.22 (d, *J* = 9.0 Hz, 1 H), 7.09 (t, *J* = 4.8 Hz, 1 H), 7.01 (d, *J* = 2.5 Hz, 1 H), 6.87–6.84 (m, 1 H), 6.43 (s, 1 H), 3.87 (s, 3 H), 3.21–3.17 (m, 2 H), 0.87–0.83 (m, 2 H), 0.03 (s, 9 H); ^{13}C NMR (100 MHz, CDCl$_3$): δ 158.3, 157.9, 155.3, 145.8, 132.0, 130.1, 116.6, 114.9, 111.1, 105.1, 102.2, 55.7, 24.1, 16.6, −1.8; HRMS (ESI) Calcd for $C_{18}H_{23}N_3OSi$ [M + H]$^+$ 326.1689, found 326.1690.

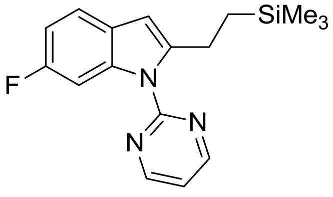

6-Fluoro-1-(pyrimidin-2-yl)-2-(2-(trimethylsilyl)ethyl)-1*H*-indole (3ea): 52% yield; colorless oil; R_f 0.47 (hexane/EtOAc = 10/1); ^{1}H NMR (400 MHz, $CDCl_3$): δ 8.78 (d, J = 4.8 Hz, 2 H), 8.08–8.04 (m, 1 H), 7.44–40 (m, 1 H), 7.15 (t, J = 4.8 Hz, 1 H), 6.97–6.92 (m, 1 H), 6.46 (s, 1 H), 3.19–3.15 (m, 2 H), 0.85–0.81 (m, 2 H), 0.02 (s, 9 H); ^{13}C NMR (100 MHz, $CDCl_3$): δ 160.0 (d, $^1J_{C\text{-}F}$ = 235 Hz), 158.2, 158.1, 145.5 (d, $^4J_{C\text{-}F}$ = 3.8 Hz), 137.1 (d, $^3J_{C\text{-}F}$ = 12.5 Hz), 125.6, 119.9 (d, $^3J_{C\text{-}F}$ = 9.9 Hz), 117.1, 109.8 (d, $^2J_{C\text{-}F}$ = 24.0 Hz), 104.8, 101.3 (d, $^2J_{C\text{-}F}$ = 28.4 Hz), 24.0, 16.5, −1.8; HRMS (ESI) Calcd for $C_{17}H_{20}FN_3Si$ $[M + H]^+$ 314.1489, found 314.1494.

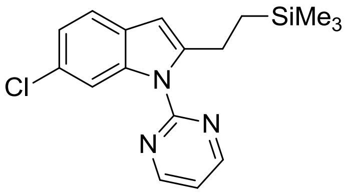

6-Chloro-1-(pyrimidin-2-yl)-2-(2-(trimethylsilyl)ethyl)-1*H*-indole (3fa): 43% yield; colorless oil; R_f 0.44 (hexane/EtOAc = 10/1); ^{1}H NMR (400 MHz, $CDCl_3$): δ 8.81 (d, J = 4.8 Hz, 2 H), 8.29 (s, 1 H), 7.41 (d, J = 8.3 Hz, 1 H), 7.20–7.13 (m, 2 H), 6.45 (s, 1 H), 3.18–3.13 (m, 2 H), 0.83–0.79 (m, 2 H), 0.01 (s, 9 H); ^{13}C NMR (100 MHz, $CDCl_3$): δ 158.2, 158.1, 145.9, 137.4, 128.1, 127.9, 122.2, 120.2, 117.3, 114.1, 104.8, 23.9, 16.5, −1.8; HRMS (ESI) Calcd for $C_{17}H_{20}ClN_3Si$ $[M + H]^+$ 330.1193, found 330.1192.

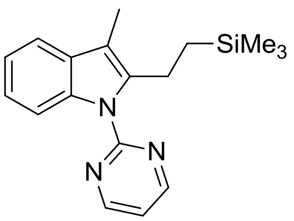

3-Methyl-1-(pyrimidin-2-yl)-2-(2-(trimethylsilyl)ethyl)-1*H*-indole (3ga): 42% yield; white solid; R_f 0.42 (hexane/EtOAc = 10/1); ^{1}H NMR (400 MHz, $CDCl_3$): δ 8.77 (d, J = 4.8 Hz, 2 H), 8.26–8.24 (m, 1 H), 7.50–7.48 (m, 1 H), 7.22–7.20 (m, 2 H), 7.11 (t, J = 4.8 Hz, 1 H), 3.18–3.14 (m, 2 H), 2.29 (s, 3 H), 0.73–0.69 (m, 2 H), 0.01 (s, 9 H); ^{13}C NMR (100 MHz, $CDCl_3$): δ 158.4, 158.0, 139.9, 136.1, 130.6, 122.5, 121.4, 117.8, 116.6, 113.5, 111.6, 20.4, 17.3, 8.7, −1.9; HRMS (ESI) Calcd for $C_{18}H_{23}N_3Si$ $[M + H]^+$ 310.1740, found 310.1743.

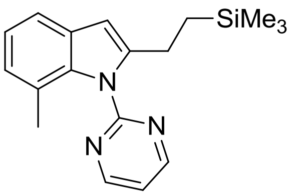

7-Methyl-1-(pyrimidin-2-yl)-2-(2-(trimethylsilyl)ethyl)-1*H*-indole (3ha): 71% yield; colorless oil; R_f 0.19 (hexane/EtOAc = 10/1); ^{1}H NMR (400 MHz, $CDCl_3$): δ 8.86 (d, *J* = 4.8 Hz, 2 H), 7.45 (d, *J* = 7.7 Hz, 1 H), 7.28 (t, *J* = 4.8 Hz, 1 H), 7.09 (t, *J* = 7.4 Hz, 1 H), 6.95 (d, *J* = 7.2 Hz, 1 H), 6.46 (s, 1 H), 2.74–2.69 (m, 2 H), 1.96 (s, 3 H), 0.82–0.78 (m, 2 H), −0.02 (s, 9 H); ^{13}C NMR (100 MHz, $CDCl_3$): δ 159.0, 158.3, 144.9, 136.6, 129.6, 124.7, 121.4, 121.0, 119.0, 117.8, 102.3, 21.8, 19.9, 15.8, −1.9; HRMS (ESI) Calcd for $C_{18}H_{23}N_3Si$ $[M + H]^+$ 310.1740, found 310.1738.

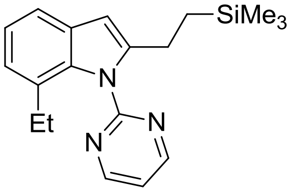

7-Ethyl-1-(pyrimidin-2-yl)-2-(2-(trimethylsilyl)ethyl)-1*H*-indole (3ia): 58% yield; colorless oil; R_f 0.27 (hexane/EtOAc = 10/1); ^{1}H NMR (400 MHz, $CDCl_3$): δ 8.86 (d, *J* = 4.8 Hz, 2 H), 7.44 (d, *J* = 6.9 Hz, 1 H), 7.30 (t, *J* = 4.8 Hz, 1 H), 7.13 (t, *J* = 7.5 Hz, 1 H), 7.02 (d, *J* = 7.3 Hz, 1 H), 6.47 (s, 1 H), 2.70–2.65 (m, 2 H), 2.30 (q, *J* = 7.5 Hz, 2 H), 0.98 (t, *J* = 7.5 Hz, 3 H), 0.81–0.76 (m, 2 H), −0.04 (s, 9 H); ^{13}C NMR (100 MHz, $CDCl_3$): δ 159.5, 158.3, 145.1, 136.0, 129.9, 127.8, 122.5, 121.2, 119.2, 117.9, 102.5, 25.7, 21.9, 15.8, 13.9, −1.9; HRMS (ESI) Calcd for $C_{19}H_{25}N_3Si$ $[M + H]^+$ 324.1896, found 324.1892.

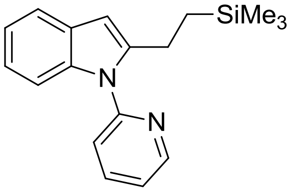

1-(Pyridin-2-yl)-2-(2-(trimethylsilyl)ethyl)-1*H*-indole (3ka): 80% yield; colorless oil; R_f 0.42 (hexane/EtOAc = 10/1); ^{1}H NMR (400 MHz, $CDCl_3$): δ 8.69–8.67 (m, 1 H), 7.91–7.87 (m, 1 H), 7.62–7.60 (m, 1 H), 7.46 (d, *J* = 8.0 Hz, 1 H), 7.38–7.31 (m, 2 H), 7.17–7.14 (m, 2 H), 6.50

(s, 1 H), 2.92–2.88 (m, 2 H), 0.82–0.78 (m, 2 H), −0.02 (s, 9 H); ^{13}C NMR (100 MHz, $CDCl_3$): δ 151.6, 149.5, 144.2, 138.2, 137.3, 128.6, 121.9, 121.5, 120.9, 120.5, 119.9, 109.9, 101.7, 21.9, 15.9, −1.9; HRMS (ESI) Calcd for $C_{18}H_{22}N_2Si$ $[M + H]^+$ 295.1631, found 295.1631.

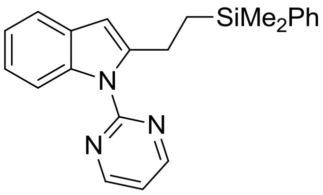

2-(2-(Dimethyl(phenyl)silyl)ethyl)-1-(pyrimidin-2-yl)-1*H*-indole (3ab): 50% yield; colorless oil; R_f 0.33 (hexane/EtOAc = 10/1); ^{1}H NMR (400 MHz, $CDCl_3$): δ 8.70 (d, J = 4.8 Hz, 2 H), 8.29 (d, J = 8.0 Hz, 1 H), 7.57–7.55 (m, 3 H), 7.42–7.39 (m, 3 H), 7.26–7.22 (m, 2 H), 7.09 (t, J = 4.8 Hz, 1 H), 6.52 (s, 1 H), 3.27–3.23 (m, 2 H), 1.16–1.12 (m, 2 H), 0.34 (s, 6 H); ^{13}C NMR (100 MHz, $CDCl_3$): δ 158.2, 158.0, 144.7, 139.0, 137.0, 133.6, 129.3, 128.9, 127.7, 122.4, 121.7, 119.6, 116.9, 113.8, 105.2, 23.9, 15.9, −3.2; HRMS (ESI) Calcd for $C_{22}H_{23}N_3Si$ $[M + H]^+$ 358.1740, found 358.1738.

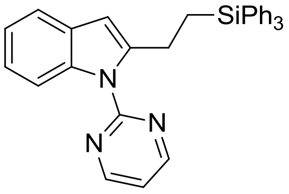

1-(Pyrimidin-2-yl)-2-(2-(triphenylsilyl)ethyl)-1*H*-indole (3ac): 31% yield; white solid; R_f 0.25 (hexane/EtOAc = 10/1); ^{1}H NMR (400 MHz, $CDCl_3$): δ 8.60 (d, J = 4.8 Hz, 2 H), 8.36–8.34 (m, 1 H), 7.64–7.57 (m, 7 H), 7.50–7.41 (m, 9 H), 7.28–7.24 (m, 2 H), 7.07 (t, J = 4.8 Hz, 1 H), 6.58 (s, 1 H), 3.45–3.41 (m, 2 H), 1.84–1.80 (m, 2 H); ^{13}C NMR (100 MHz, $CDCl_3$): δ 158.1, 157.9, 144.6, 137.0, 135.7, 134.8, 129.5, 129.3, 127.9, 122.5, 121.8, 119.7, 116.8, 113.9, 105.3, 24.1, 13.8; HRMS (ESI) Calcd for $C_{32}H_{27}N_3Si$ $[M + H]^+$ 482.2053, found 482.2051.

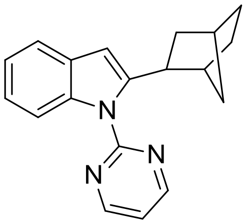

2-(Bicyclo[2.2.1]heptan-2-yl)-1-(pyrimidin-2-yl)-1*H*-indole (3ad): 30% yield; colorless oil; R_f 0.33 (hexane/EtOAc = 10/1); ^{1}H NMR (400 MHz, $CDCl_3$): δ 8.82 (d, J = 4.8 Hz, 2 H), 8.08–8.06 (m, 1 H), 7.56–7.53 (m, 1 H), 7.21–7.15 (m, 3 H), 6.49 (s, 1 H), 3.71–3.67 (m, 1 H), 2.38–2.29 (m, 2 H), 1.65–1.63 (m, 1 H), 1.58–1.53 (m, 4 H), 1.38–1.34 (m, 1 H), 1.27–1.24 (m, 1 H), 1.17–1.15 (m, 1 H); ^{13}C NMR (100 MHz, $CDCl_3$): δ 158.4, 158.2, 146.9, 137.2, 129.1, 122.3, 121.5, 119.8, 117.3, 112.9, 103.3, 42.3, 40.6, 38.1, 36.8, 36.1, 29.7, 28.9; HRMS (ESI) Calcd for $C_{19}H_{19}N_3$ $[M + H]^+$ 290.1657, found 290.1655.

Removal of the pyrimidyl group

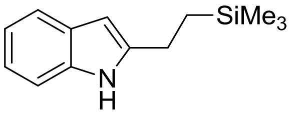

2-(2-(Trimethylsilyl)ethyl)-1*H*-indole (4aa): A mixture of 1-(pyrimidin-2-yl)-2-(2-(trimethylsilyl)ethyl)-1*H*-indole (**3aa**, 886 mg, 3 mmol) and freshly prepared sodium ethoxide (408 mg, 6 mmol) in DMSO (5 mL) was stirred at 100 ºC under nitrogen atmosphere for 20 h. After cooling to ambient temperature, the reaction mixture was extracted with EtOAc (2 × 20 mL), and the combined organic phase was dried over Na_2SO_4. After filtration and evaporation of the solvents under reduced pressure, the crude product was purified by column chromatography on silica gel (hexane/EtOAc = 50/1) to yield the title product (554 mg, 85%) as a yellow solid.

R_f 0.38 (hexane/EtOAc = 10/1); ^{1}H NMR (400 MHz, $CDCl_3$): δ 7.87 (brs, 1 H), 7.53 (d, *J* = 7.6 Hz, 1 H), 7.29 (d, *J* = 7.2 Hz, 1 H), 7.14–7.05 (m, 2 H), 6.25 (s, 1 H), 2.80–2.76 (m, 2 H), 1.01–0.96 (m, 2 H), 0.06 (s, 9 H); ^{13}C NMR (100 MHz, $CDCl_3$): δ 142.2, 135.9, 128.8, 120.9, 119.7, 119.5, 110.2, 98.8, 22.5, 15.9, –1.8; HRMS (ESI) Calcd for $C_{13}H_{19}NSi$ $[M + H]^+$ 218.1365, found 218.1363

References

1. Ackermann, L.; Lygin, A. V. *Org. Lett.* **2011**, *13*, 3332–3335.
2. Ding, Z.; Yoshikai, N. *Angew. Chem., Int. Ed.* **2012**, *51*, 4698–4701.

.

^{1}H and ^{13}C NMR spectra

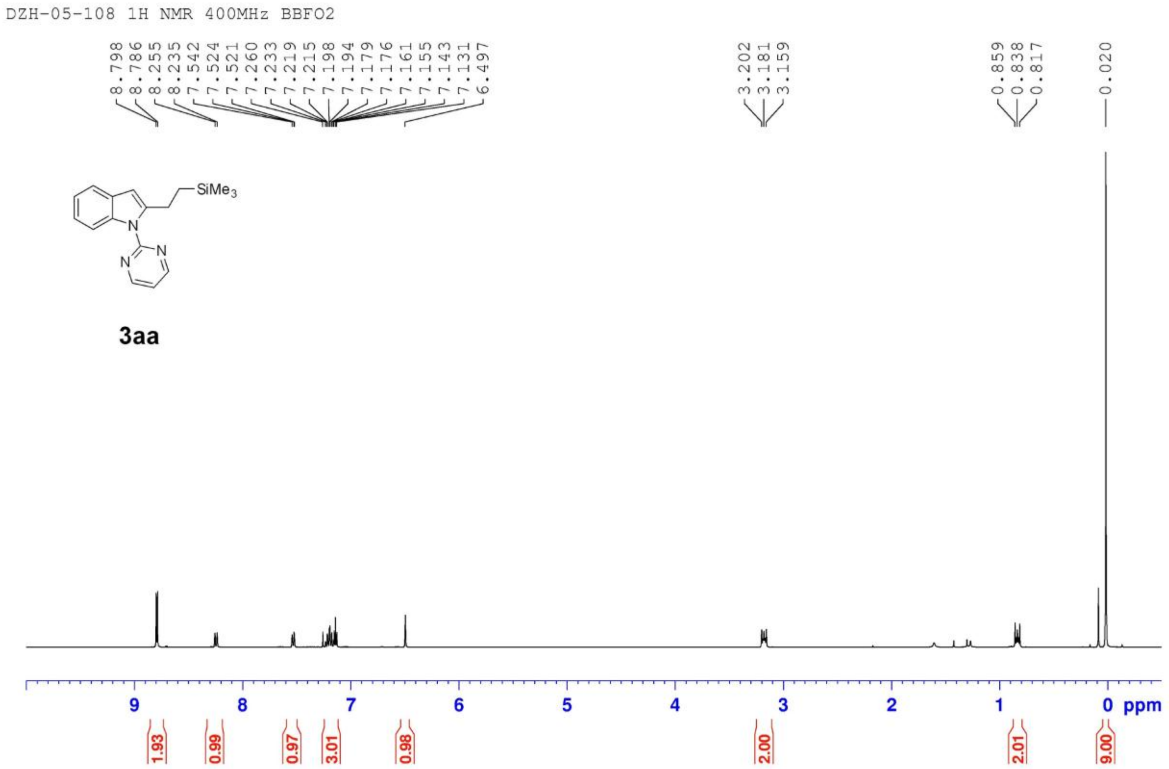

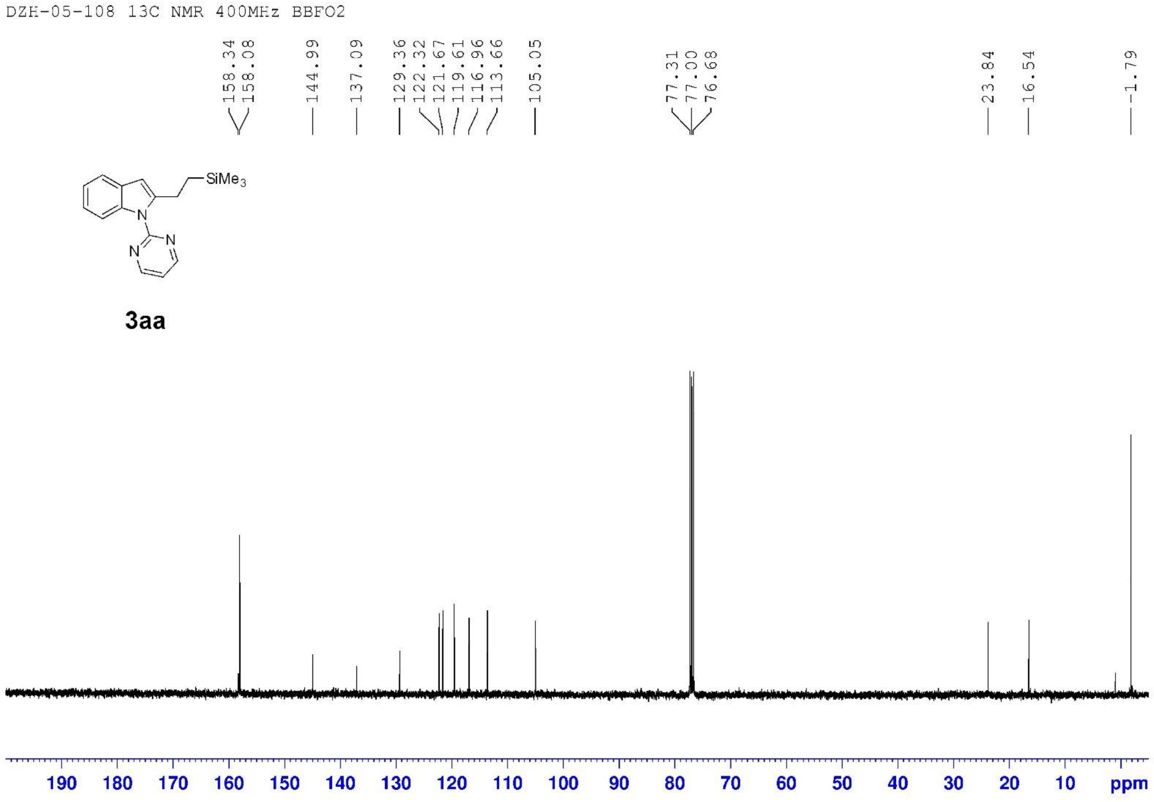

DZH-05-095 1H NMR CDCl3 BBOF1 400MHz

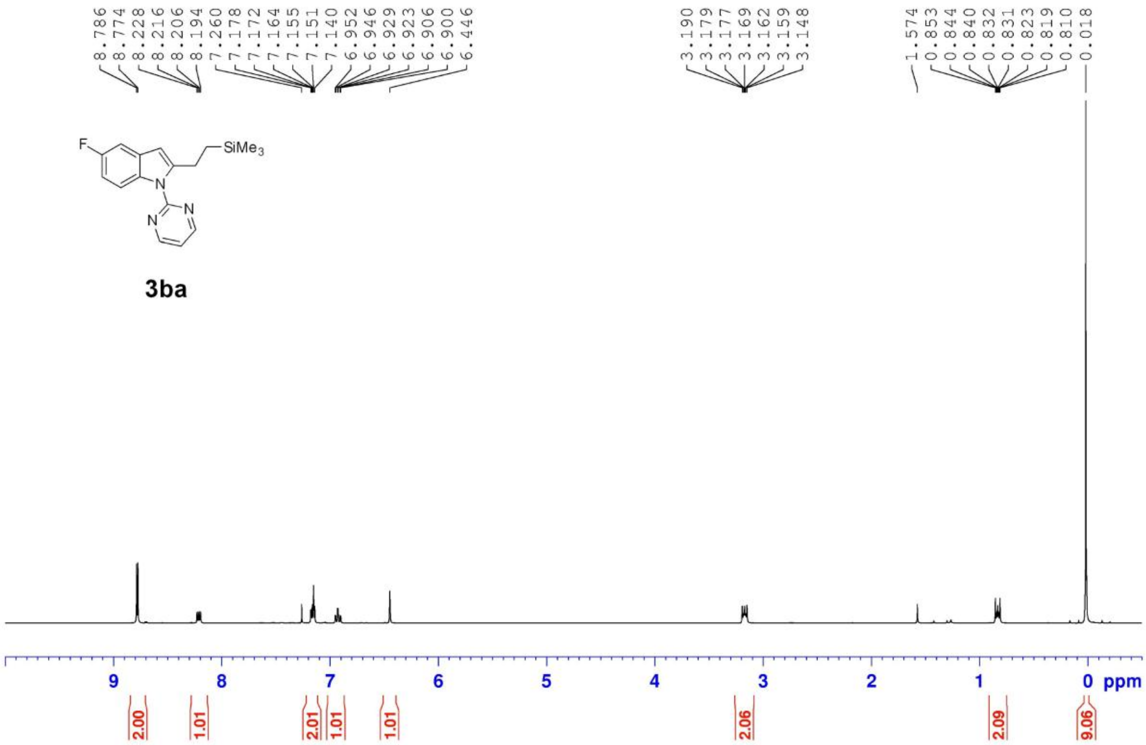

DZH-05-095 13C NMR CDCl3 BBOF1 400MHz

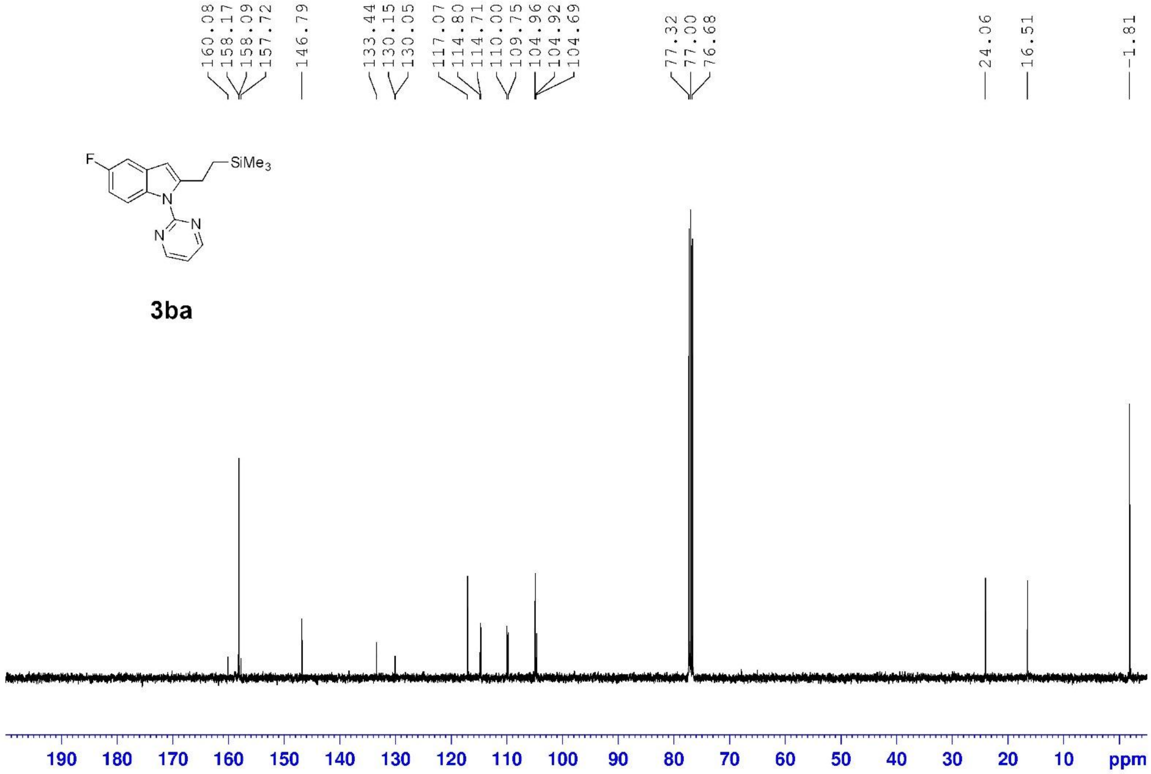

DZH-05-119 1H NMR CDCl3 BBOF1 400MHz

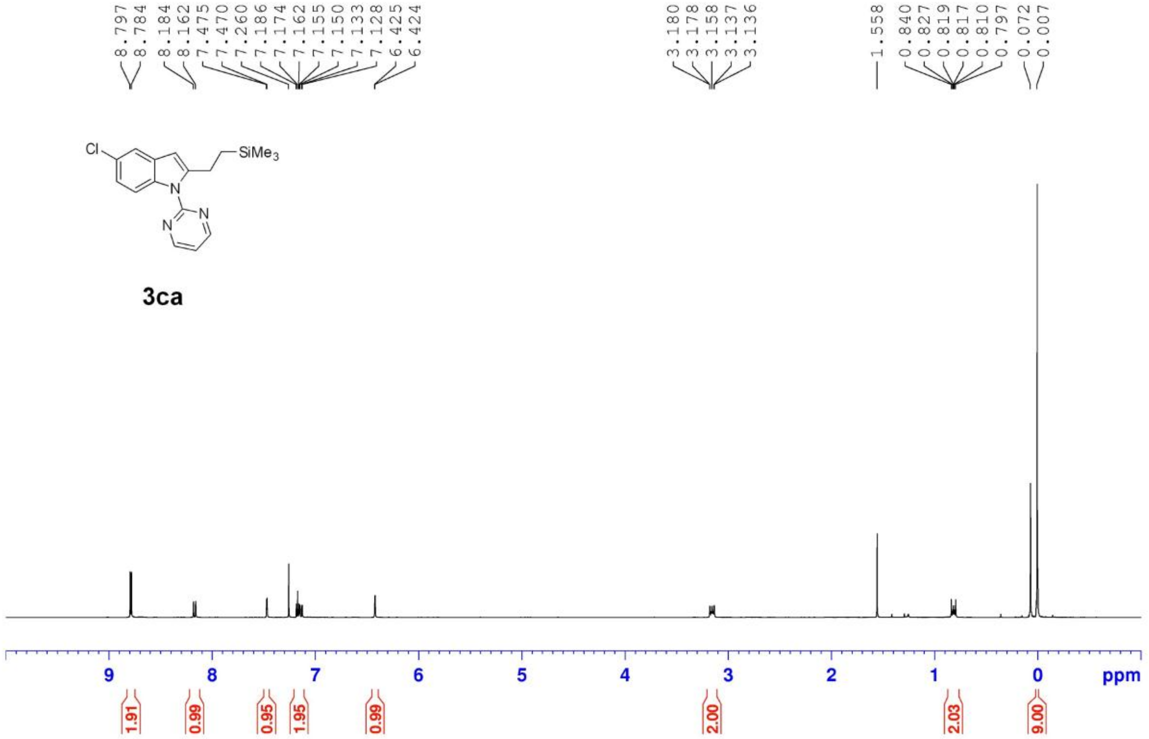

DZH-05-119 13C NMR CDCl3 BBOF1 400MHz

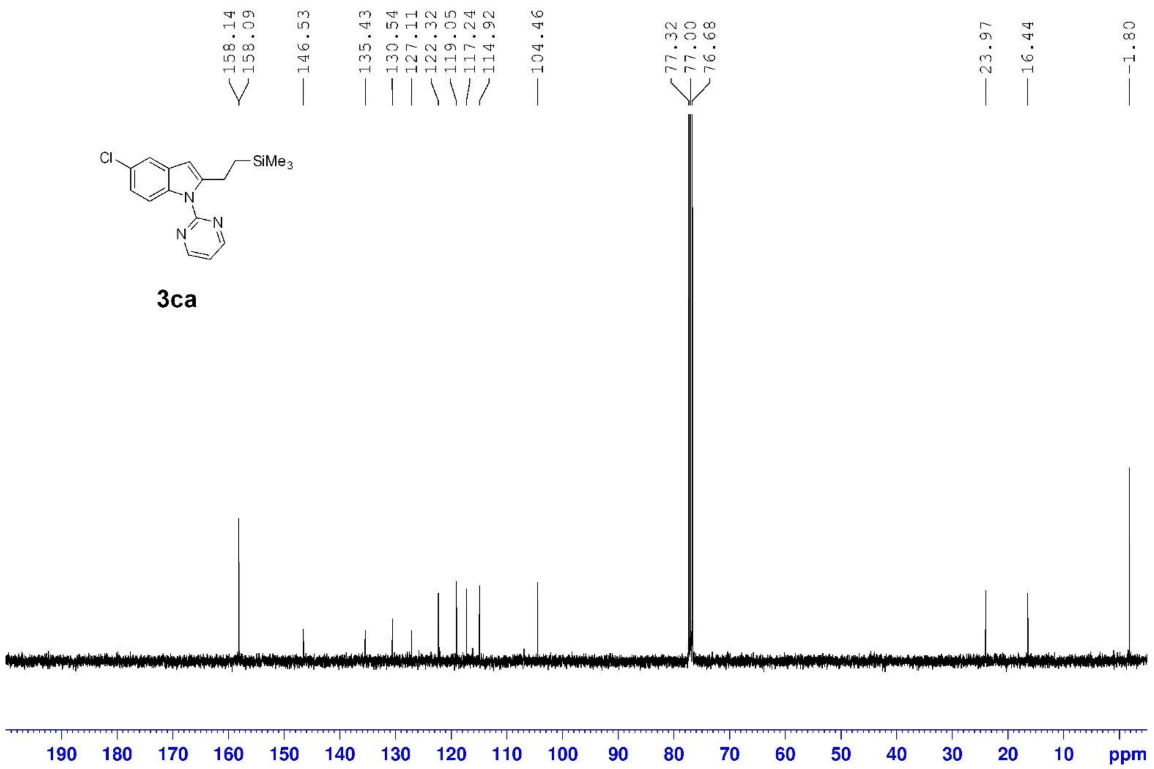

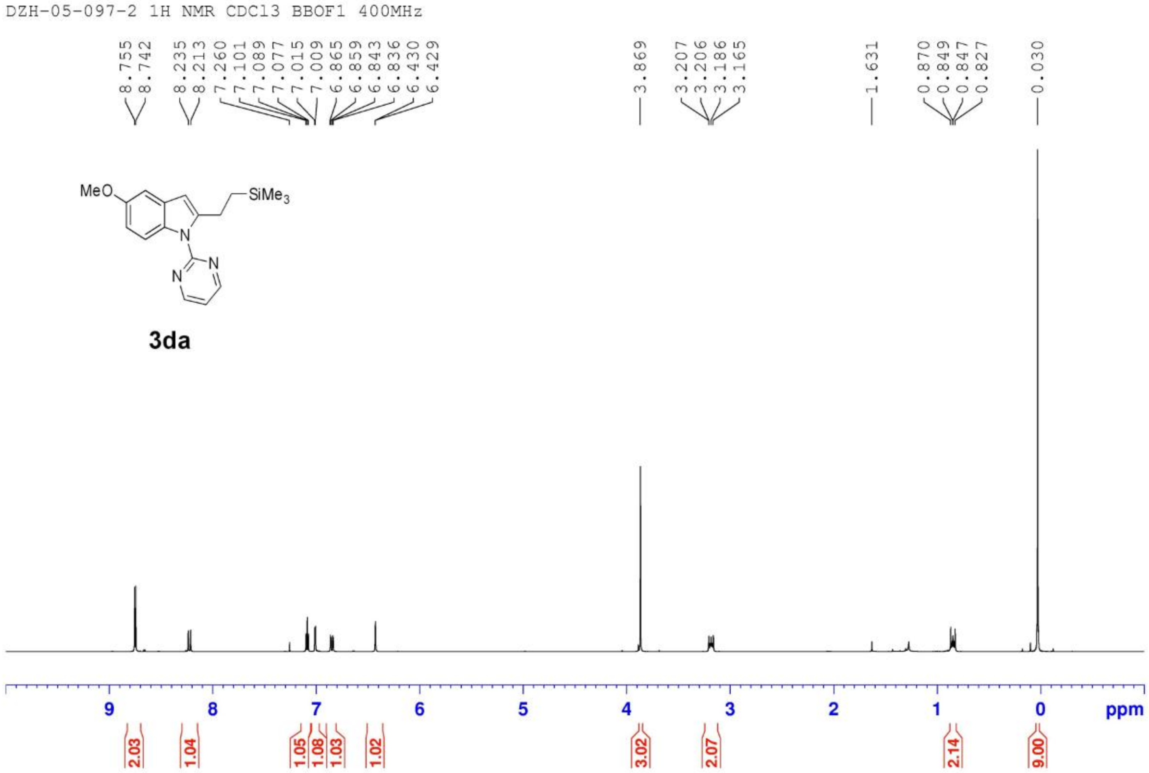
DZH-05-097-2 1H NMR CDCl3 BBOF1 400MHz
8.755
8.742
8.235
8.213
7.260
7.101
7.089
7.077
7.015
7.009
6.865
6.859
6.843
6.836
6.430
6.429
3.869
3.207
3.206
3.186
3.165
1.631
0.870
0.849
0.847
0.827
0.030
MeO
SiMe3
N
N
N
3da
9
8
7
6
5
4
3
2
1
0
ppm
2.03
1.04
1.05
1.08
1.03
1.02
3.02
2.07
2.14
9.00

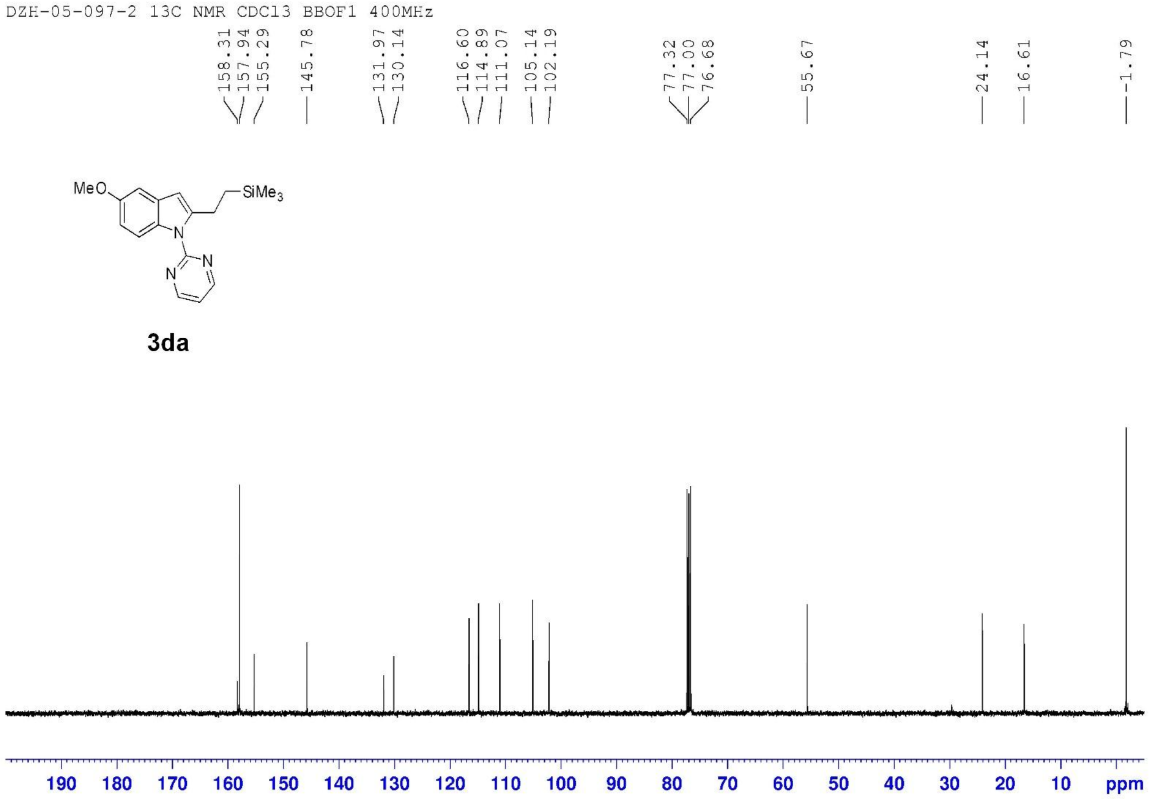
DZH-05-097-2 13C NMR CDCl3 BBOF1 400MHz
158.31
157.94
155.29
145.78
131.97
130.14
116.60
114.89
111.07
105.14
102.19
77.32
77.00
76.68
55.67
24.14
16.61
-1.79
MeO
SiMe3
N
N
N
3da
190
180
170
160
150
140
130
120
110
100
90
80
70
60
50
40
30
20
10
ppm

DZH-05-112 1H NMR CDCl3 BBOF1 400MHz

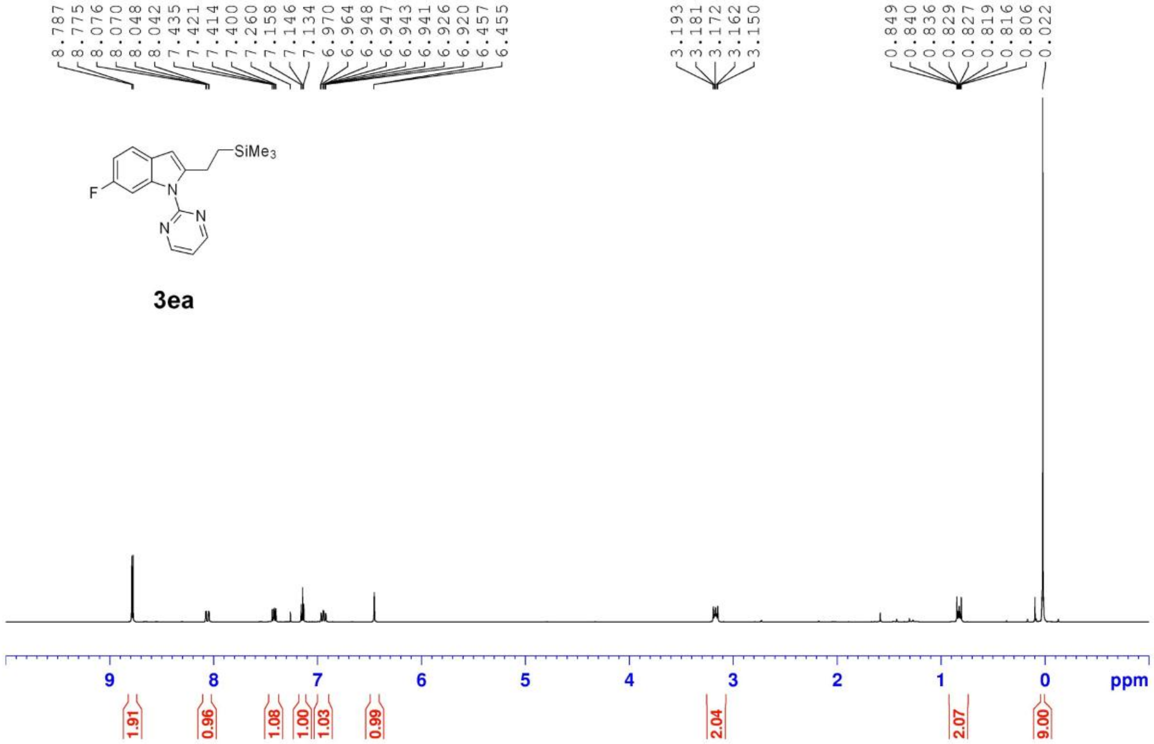

DZH-05-112 13C NMR CDCl3 BBOF1 400MHz

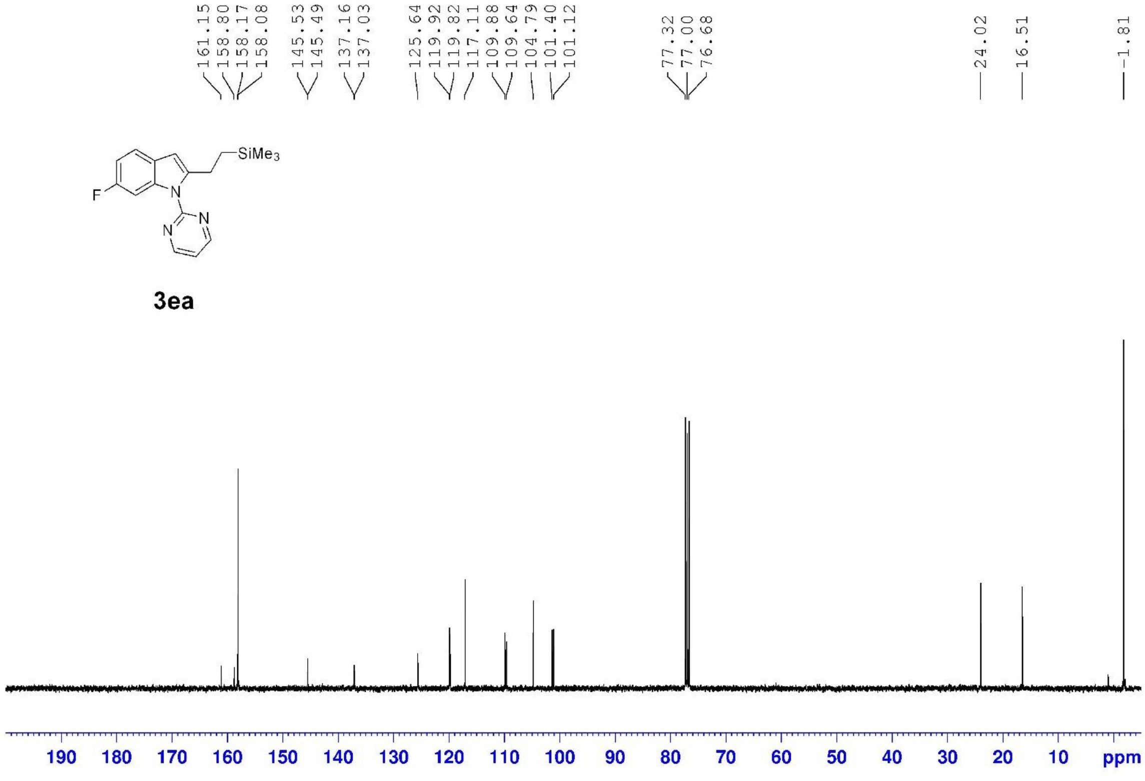

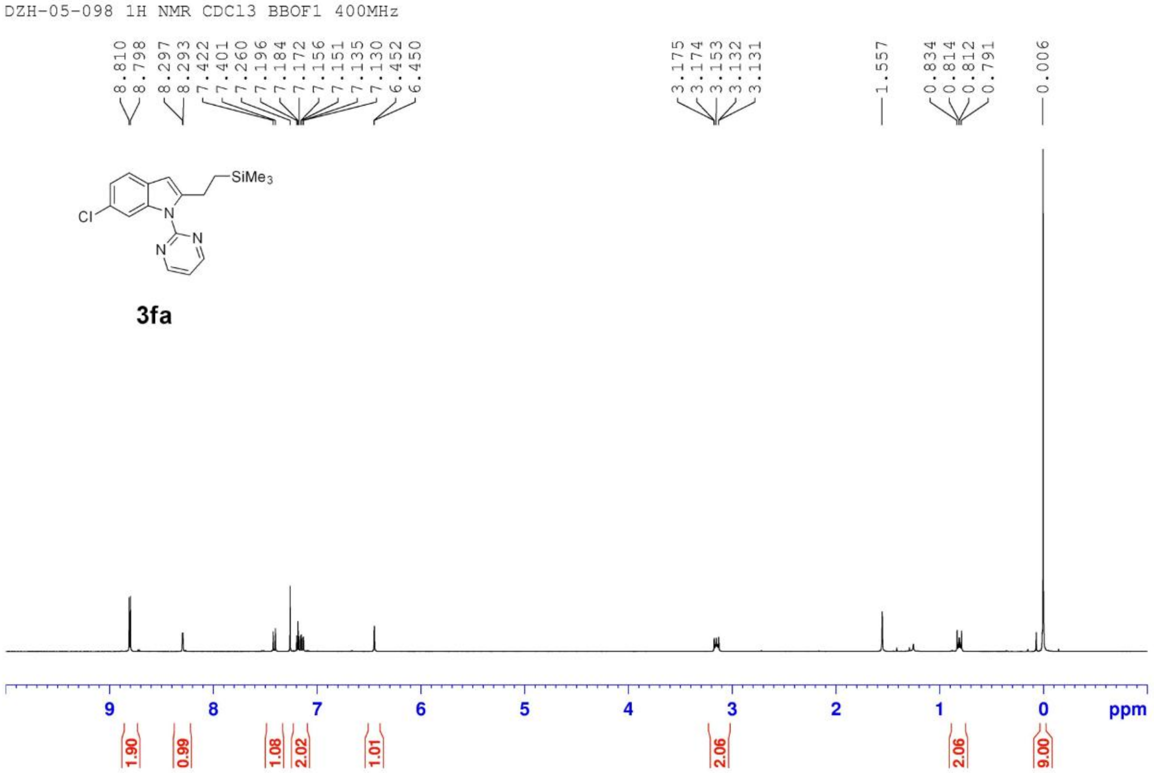
DZH-05-098 1H NMR CDCl3 BBOF1 400MHz
8.810 8.798 8.297 8.293 7.422 7.401 7.260 7.196 7.184 7.172 7.156 7.151 7.135 7.130 6.452 6.450
3.175 3.174 3.153 3.132 3.131
1.557
0.834 0.814 0.812 0.791
0.006
SiMe3
Cl
N
3fa
1.90 0.99 1.08 2.02 1.01 2.06 2.06 9.00
ppm

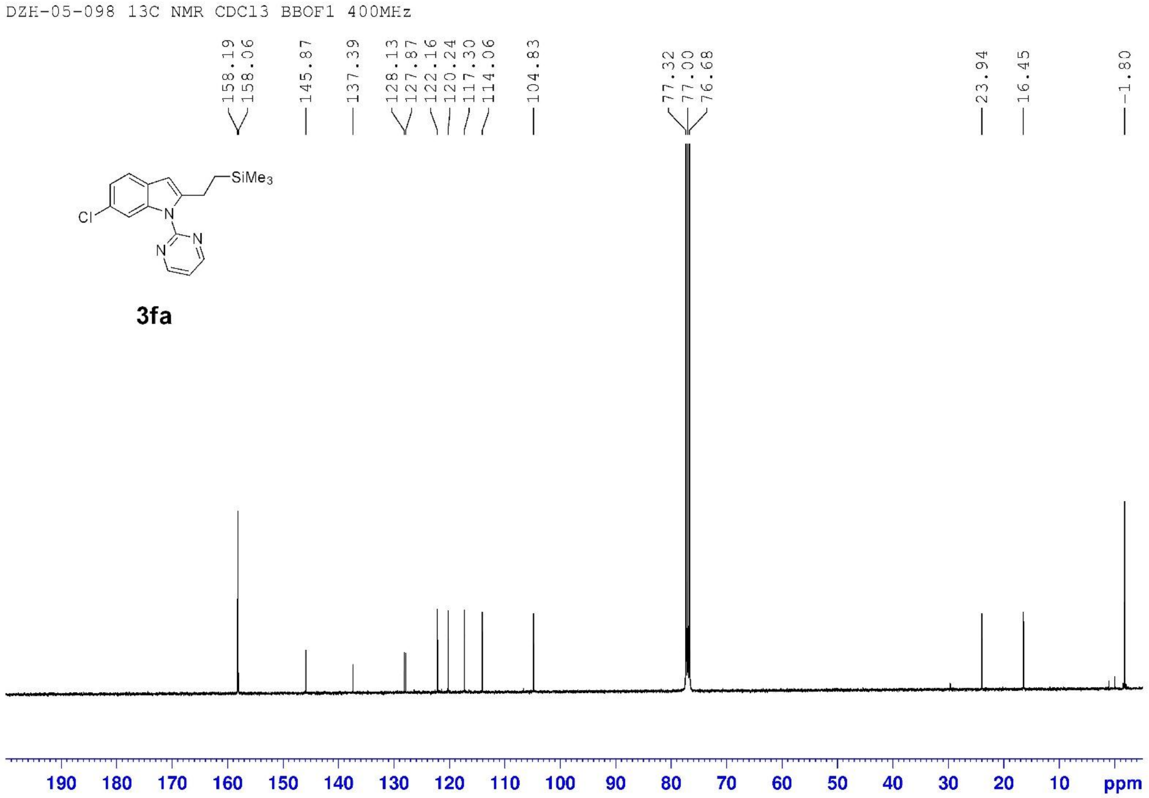
DZH-05-098 13C NMR CDCl3 BBOF1 400MHz
158.19 158.06 145.87 137.39 128.13 127.87 122.16 120.24 117.30 114.06 104.83
77.32 77.00 76.68
23.94 16.45 -1.80
SiMe3
Cl
N
3fa
ppm

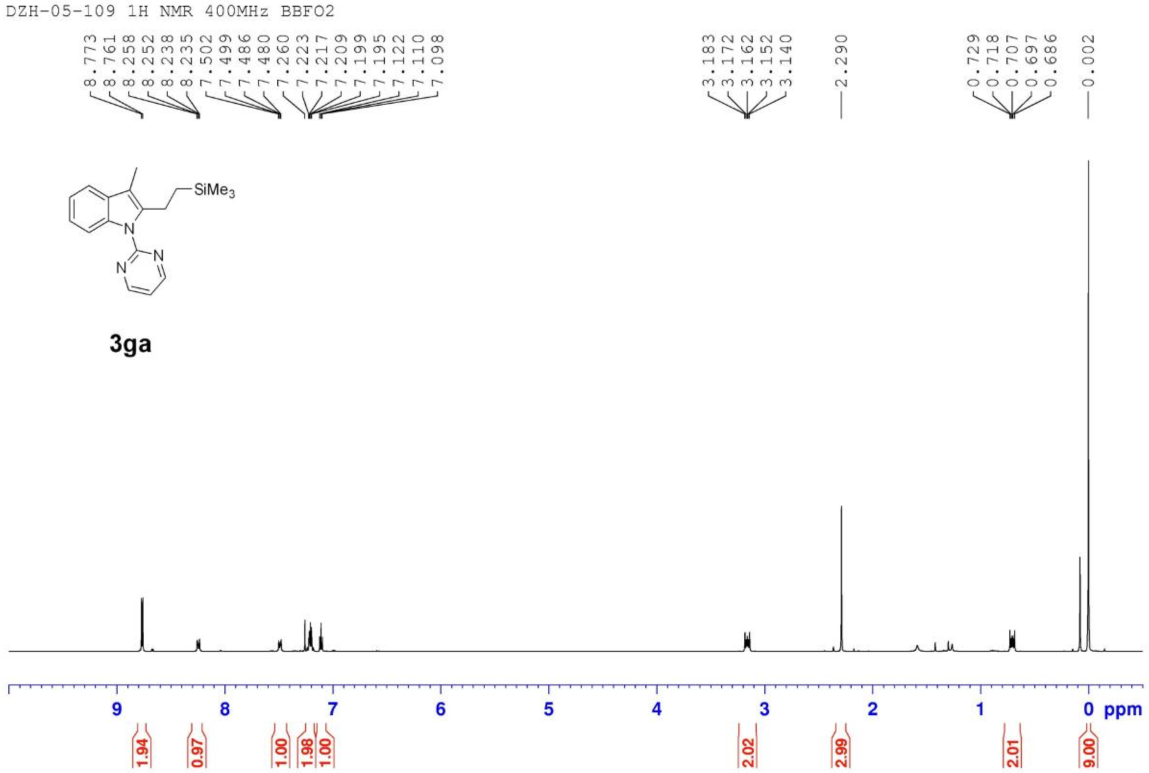
DZH-05-109 1H NMR 400MHz BBFO2
8.773 8.761 8.258 8.252 8.238 8.235 7.502 7.499 7.486 7.480 7.260 7.223 7.217 7.209 7.199 7.195 7.122 7.110 7.098
3.183 3.172 3.162 3.152 3.140
2.290
0.729 0.718 0.707 0.697 0.686
0.002
SiMe3
N
N
N
3ga
9 8 7 6 5 4 3 2 1 0 ppm
1.94 0.97 1.00 1.98 1.00 2.02 2.99 2.01 9.00

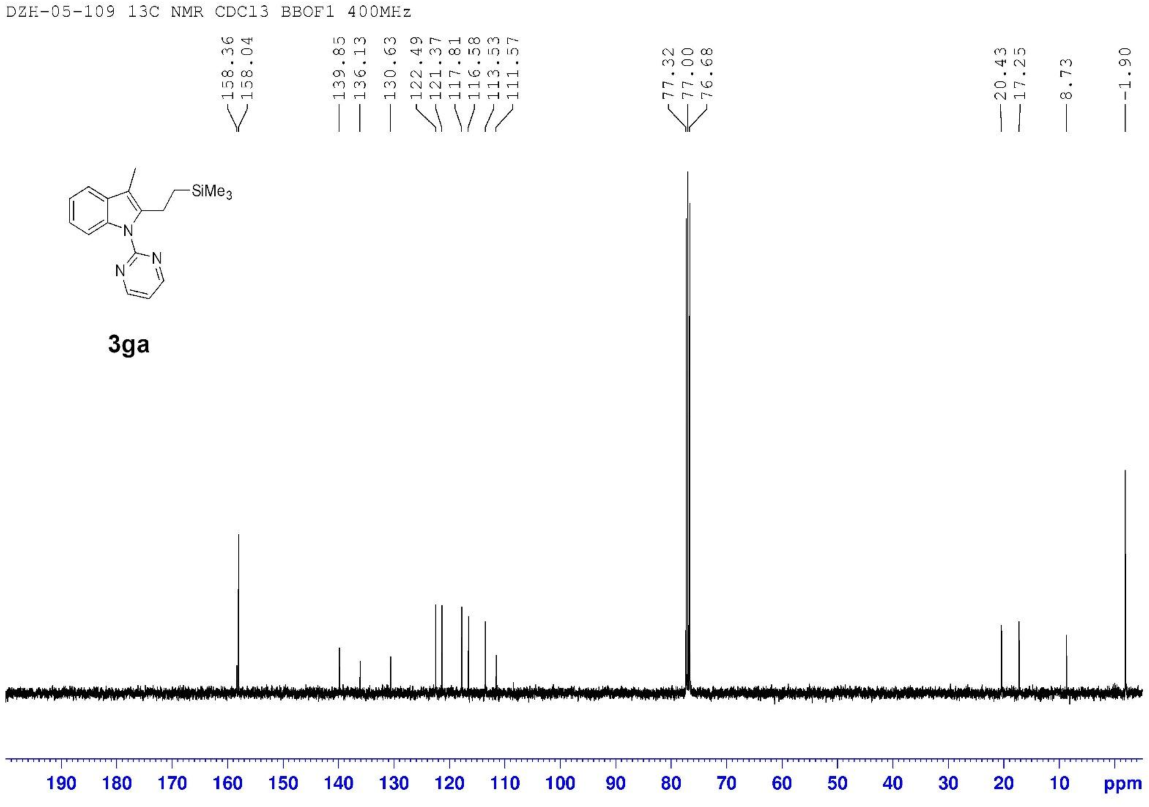
DZH-05-109 13C NMR CDCl3 BBOF1 400MHz
158.36 158.04
139.85 136.13 130.63 122.49 121.37 117.81 116.58 113.53 111.57
77.32 77.00 76.68
20.43 17.25 8.73 -1.90
SiMe3
N
N
N
3ga
190 180 170 160 150 140 130 120 110 100 90 80 70 60 50 40 30 20 10 ppm

DZH-05-128 1H NMR CDCl3 BBOF1 400MHz

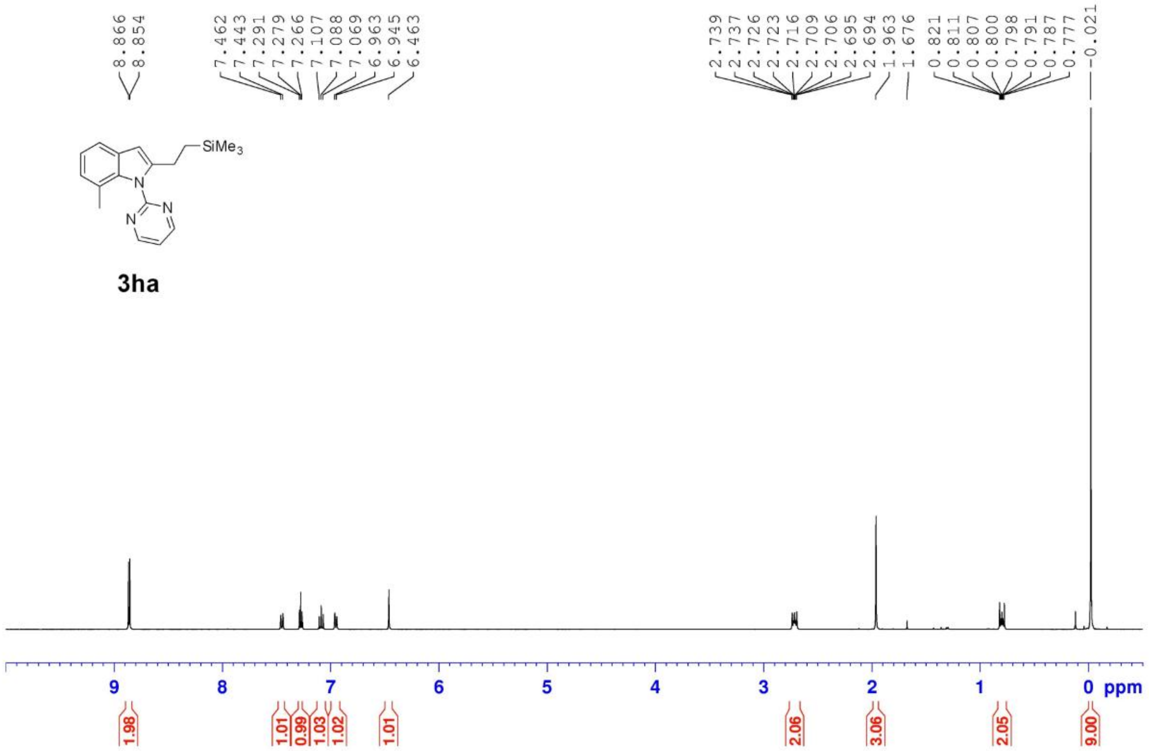

DZH-05-128 13C NMR CDCl3 BBOF1 400MHz

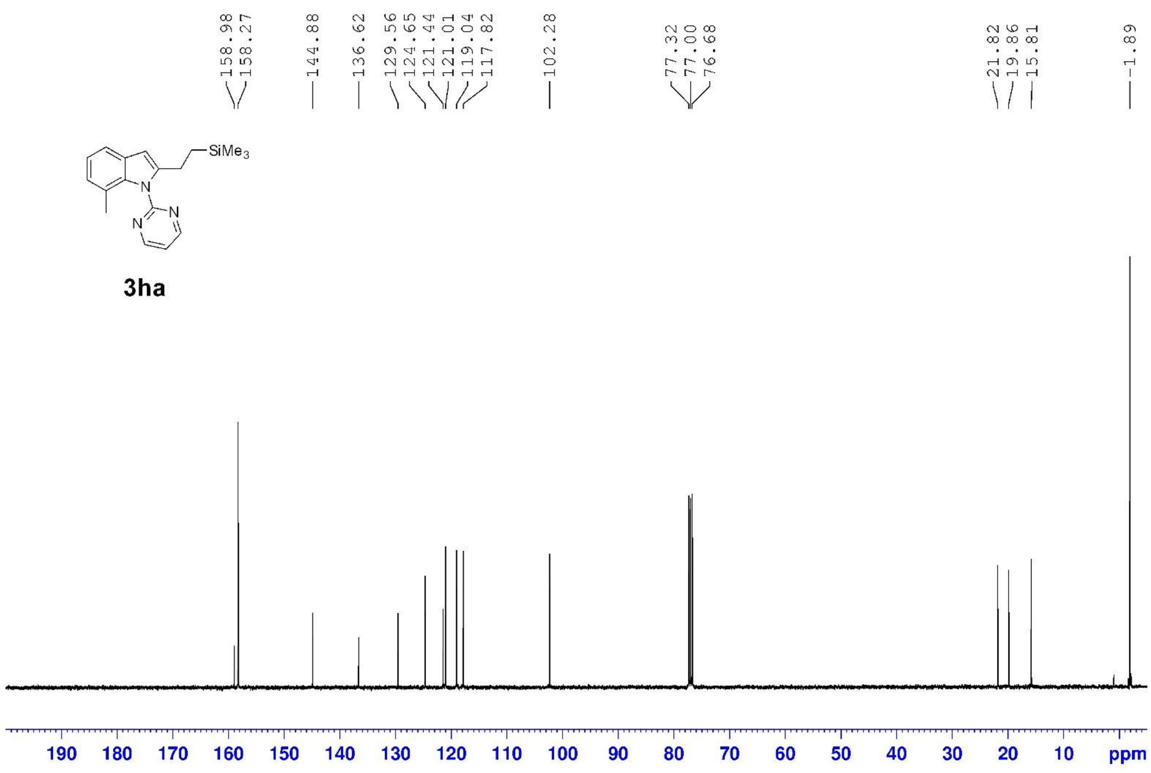

DZH-05-117 1H NMR CDCl3 BBOF1 400MHz

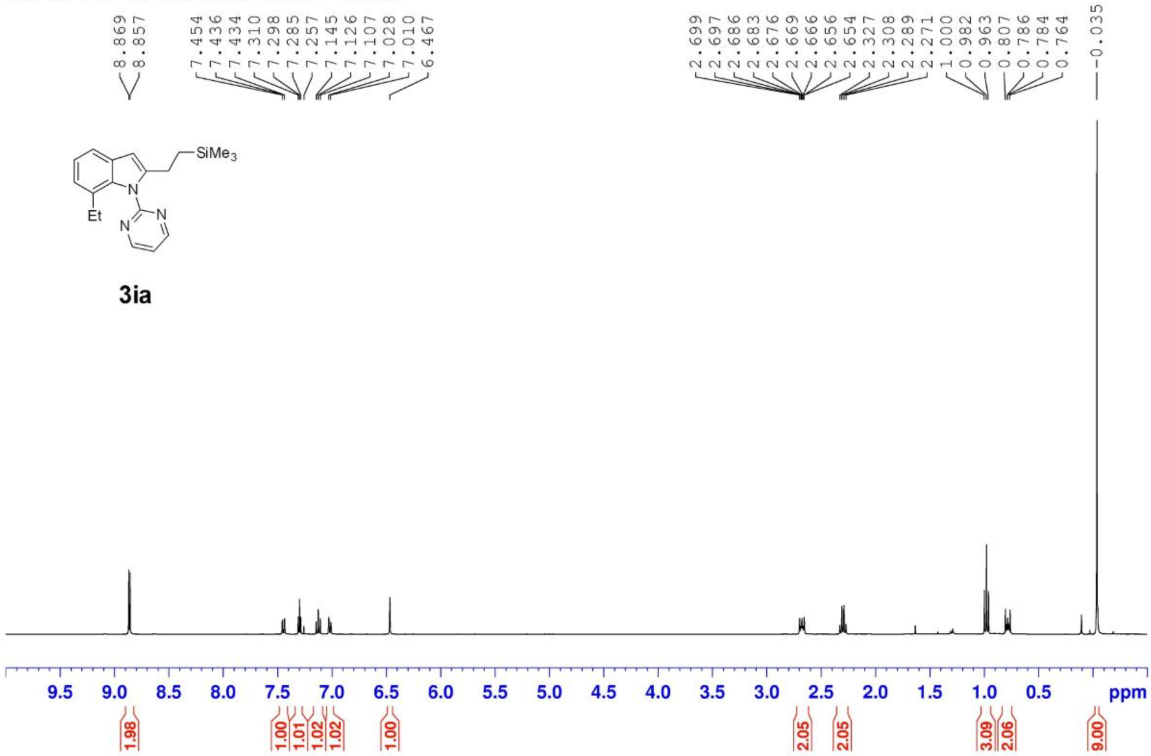

DZH-05-117 13C NMR CDCl3 BBOF1 400MHz

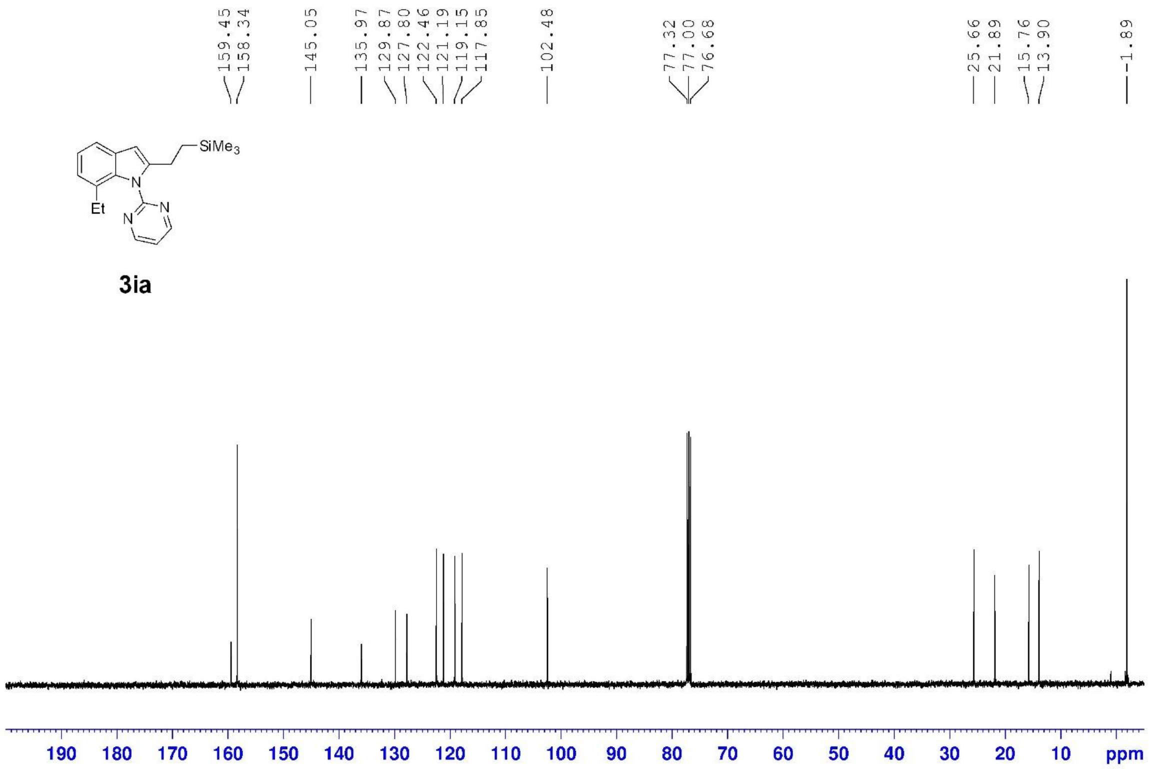

DZH-05-118 1H NMR CDCl3 BBOF1 400MHz

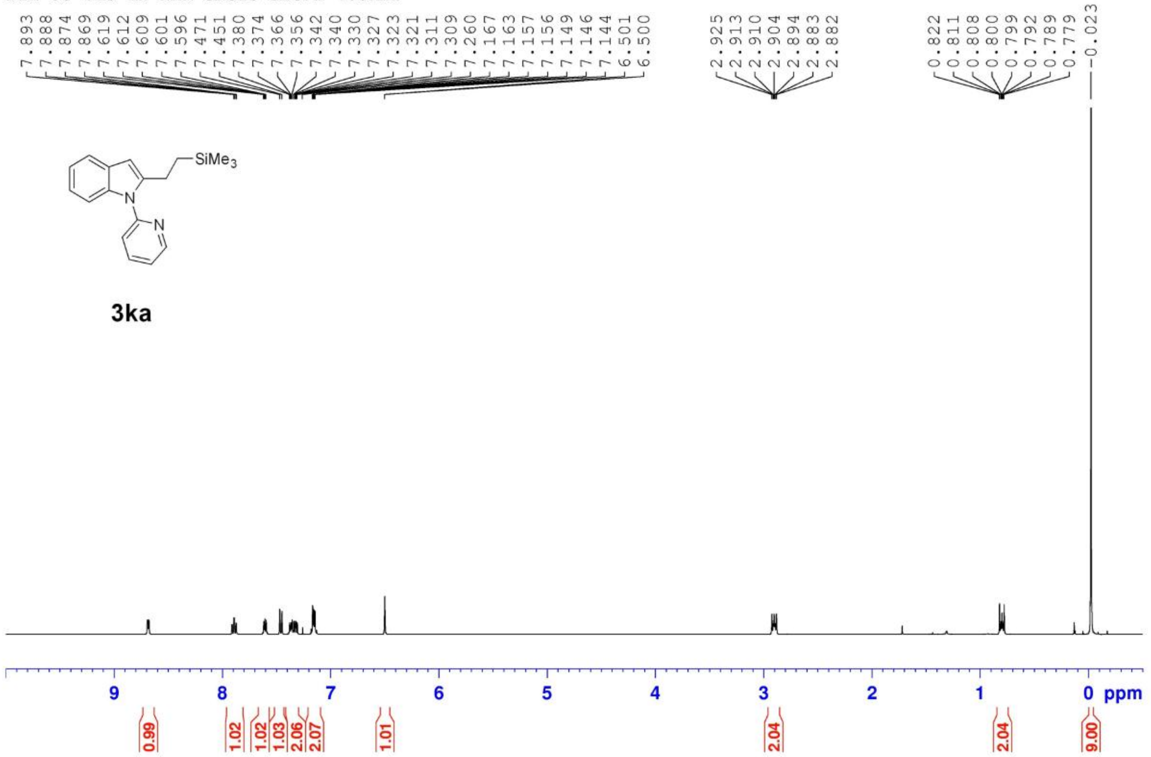

DZH-05-118 13C NMR CDCl3 BBOF1 400MHz

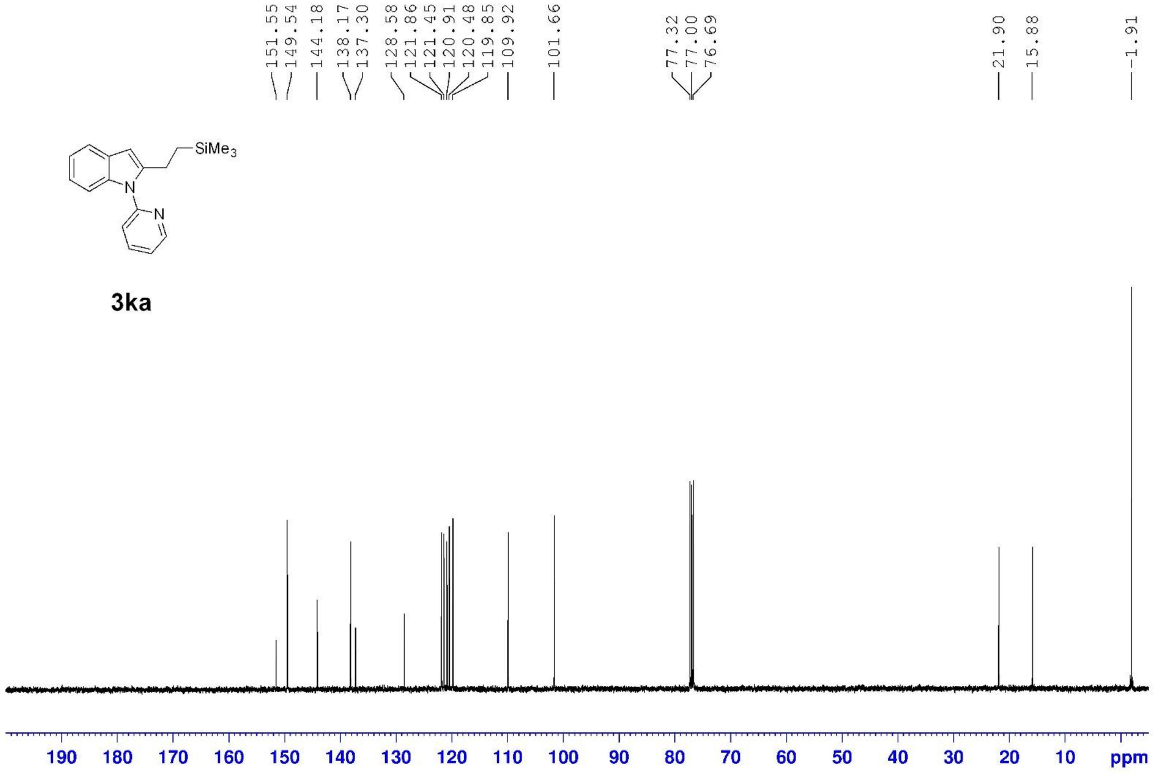

DZH-05-129 1H NMR CDCl3 BBOF1 400MHz

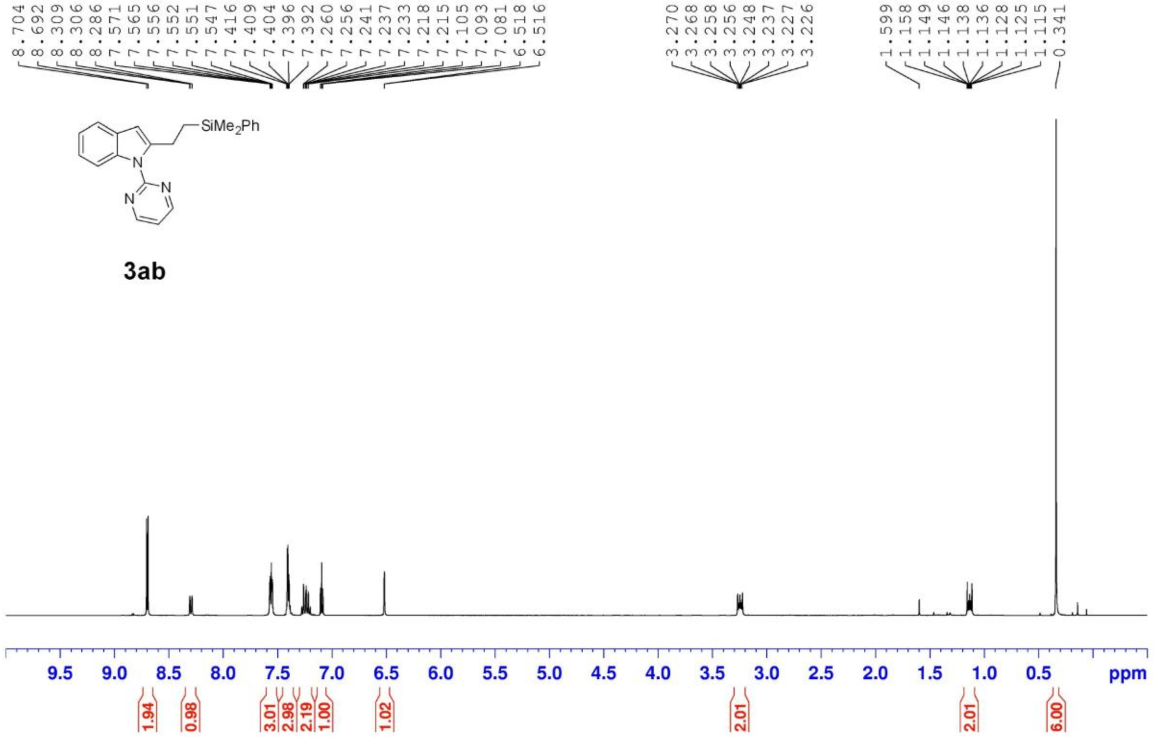

DZH-05-129 13C NMR CDCl3 BBOF1 400MHz

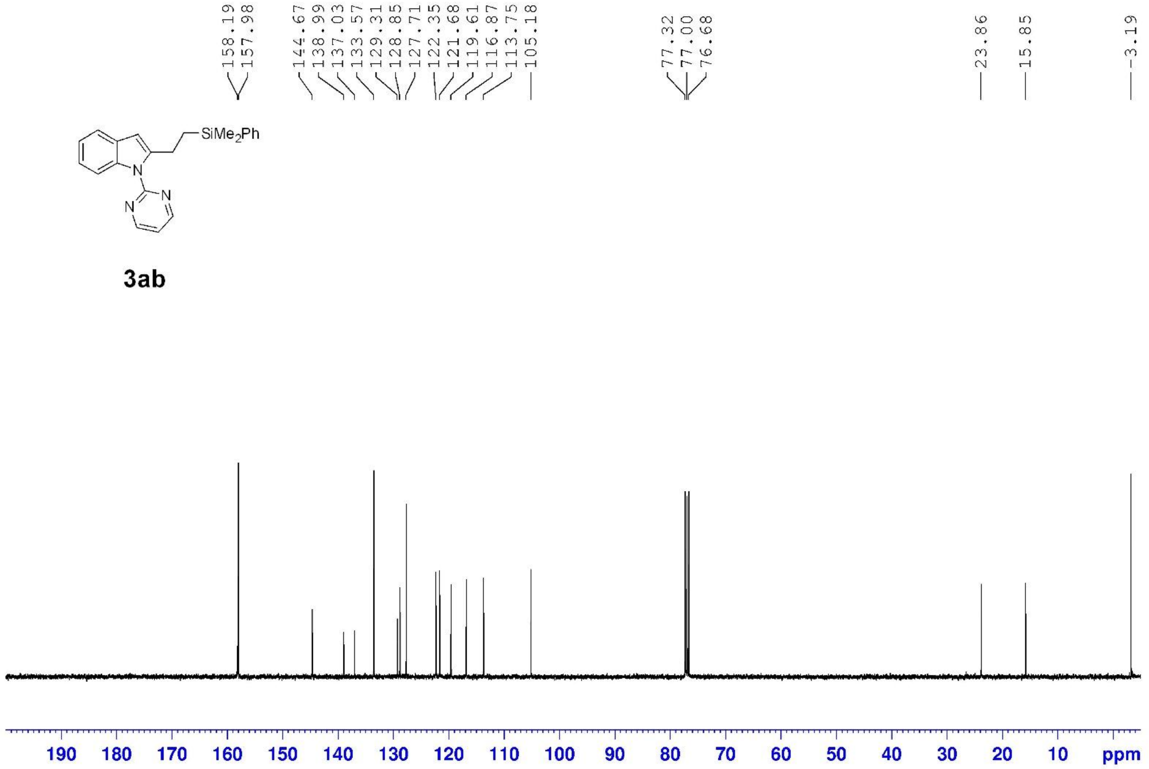

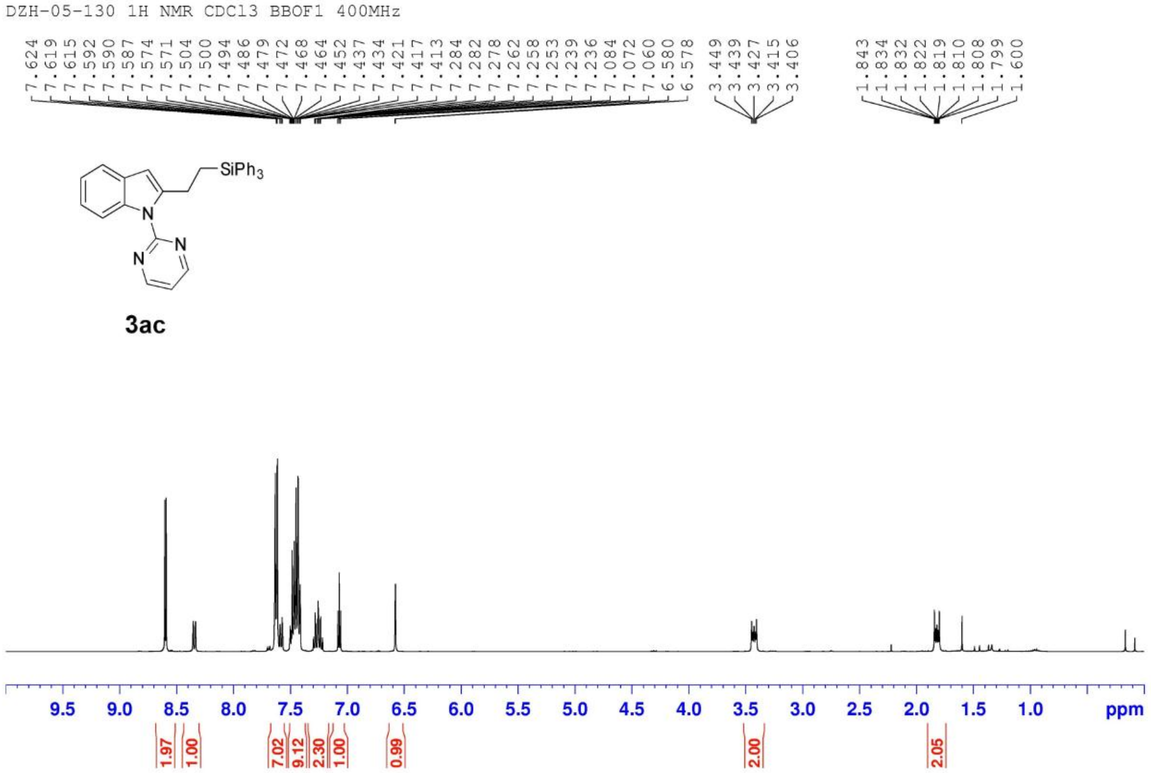
DZH-05-130 1H NMR CDCl3 BBOF1 400MHz
7.624 7.619 7.615 7.592 7.590 7.587 7.574 7.571 7.504 7.500 7.494 7.486 7.479 7.472 7.468 7.464 7.452 7.437 7.434 7.421 7.417 7.413 7.284 7.282 7.278 7.262 7.258 7.253 7.239 7.236 7.084 7.072 7.060 6.580 6.578
3.449 3.439 3.427 3.415 3.406
1.843 1.834 1.832 1.822 1.819 1.810 1.808 1.799 1.600
SiPh3
N
N
N
3ac
9.5 9.0 8.5 8.0 7.5 7.0 6.5 6.0 5.5 5.0 4.5 4.0 3.5 3.0 2.5 2.0 1.5 1.0 ppm
1.97 1.00 7.02 9.12 2.30 1.00 0.99 2.00 2.05

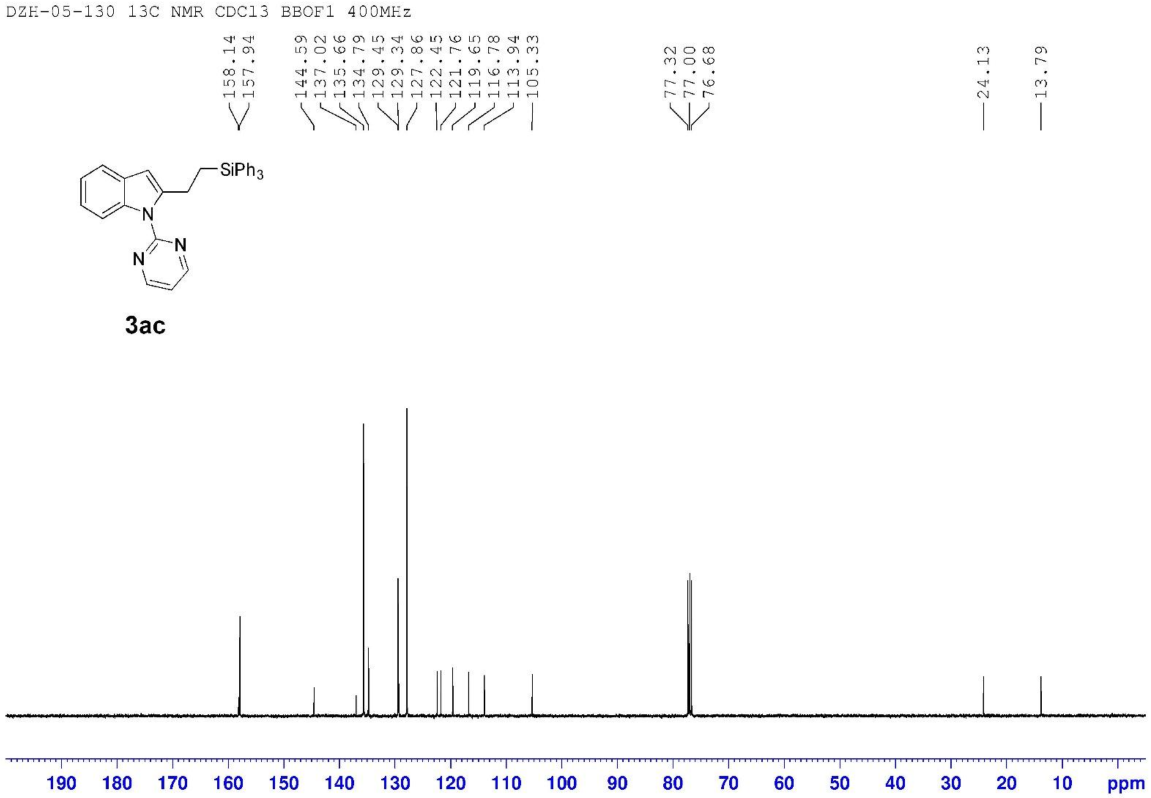
DZH-05-130 13C NMR CDCl3 BBOF1 400MHz
158.14 157.94
144.59 137.02 135.66 134.79 129.45 129.34 127.86 122.45 121.76 119.65 116.78 113.94 105.33
77.32 77.00 76.68
24.13
13.79
SiPh3
N
N
N
3ac
190 180 170 160 150 140 130 120 110 100 90 80 70 60 50 40 30 20 10 ppm

DZH-05-127 1H NMR CDCl3 BBOF1 400MHz

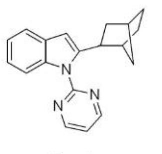

3ad

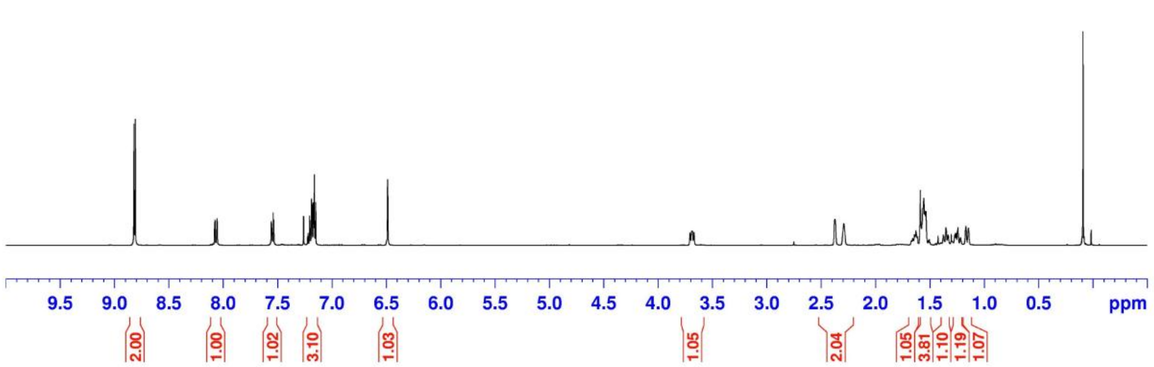

DZH-05-127 13C NMR CDCl3 BBOF1 400MHz

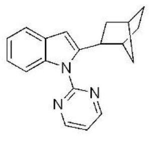

3ad

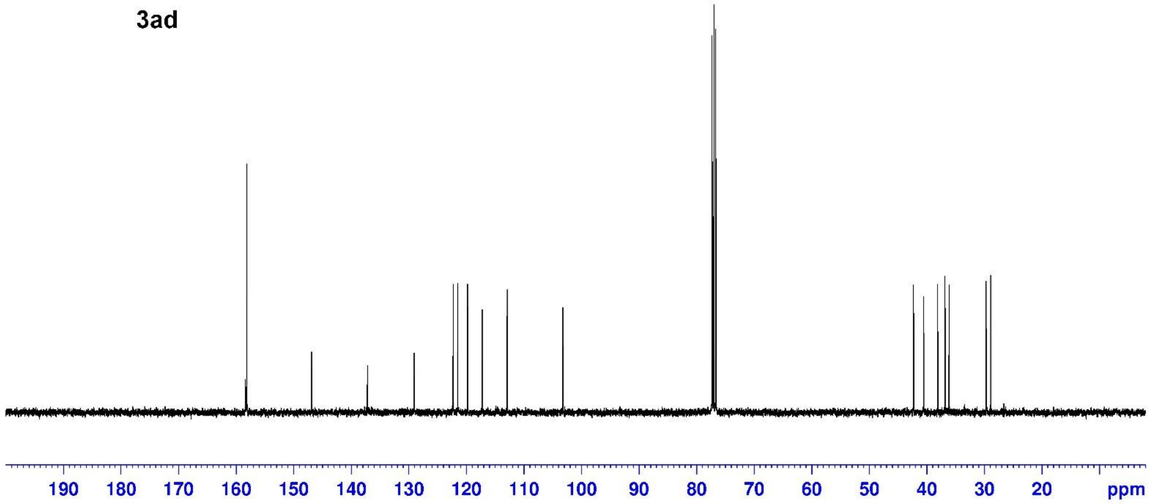

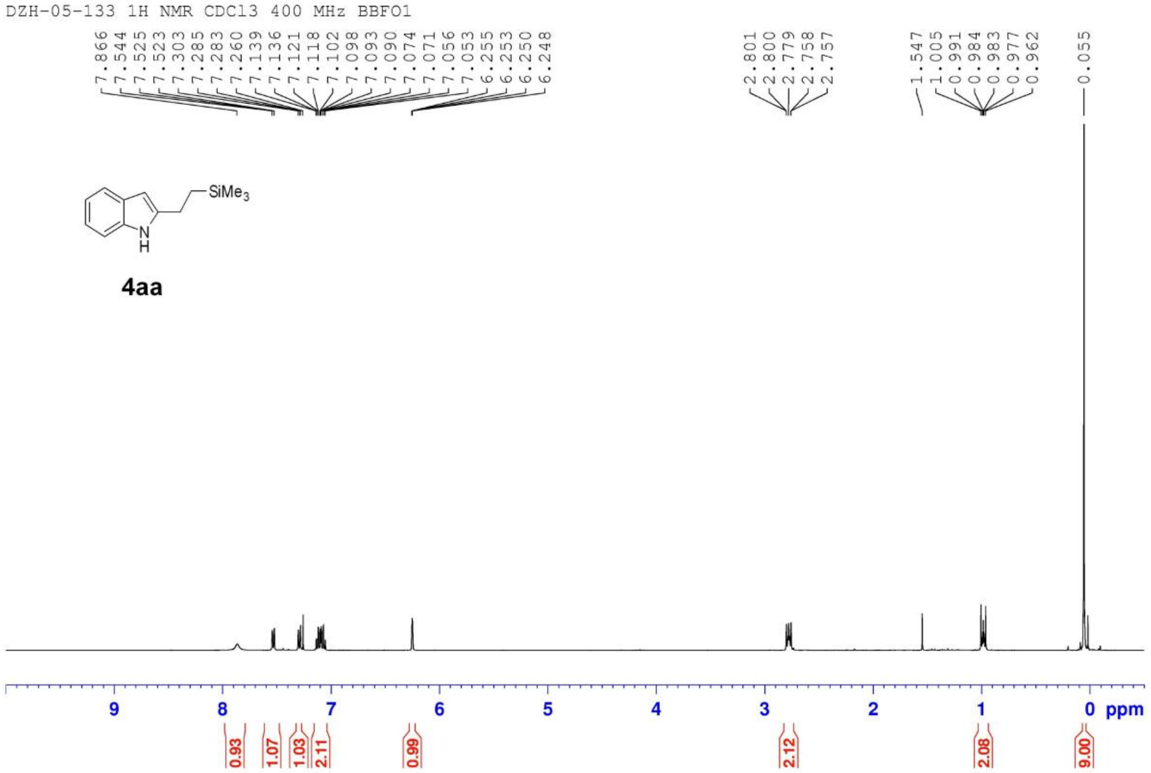
DZH-05-133 1H NMR CDCl3 400 MHz BBFO1
7.866 7.544 7.525 7.523 7.303 7.285 7.283 7.260 7.139 7.136 7.121 7.118 7.102 7.098 7.093 7.090 7.074 7.071 7.056 7.053 6.255 6.253 6.250 6.248
2.801 2.800 2.779 2.758 2.757
1.547 1.005 0.991 0.984 0.983 0.977 0.962
0.055
SiMe3
H
4aa
9 8 7 6 5 4 3 2 1 0 ppm
0.93 1.07 1.03 2.11 0.99 2.12 2.08 9.00

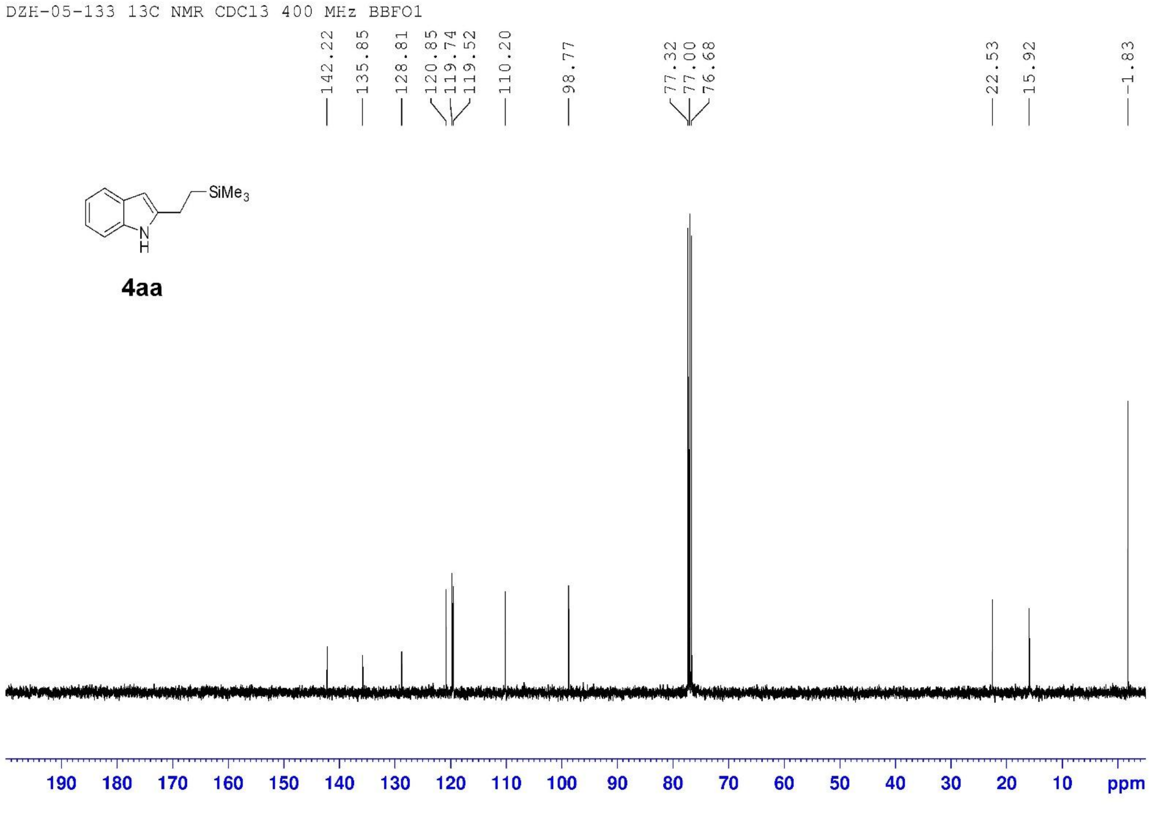
DZH-05-133 13C NMR CDCl3 400 MHz BBFO1
142.22 135.85 128.81 120.85 119.74 119.52 110.20 98.77
77.32 77.00 76.68
22.53 15.92 -1.83
SiMe3
H
4aa
190 180 170 160 150 140 130 120 110 100 90 80 70 60 50 40 30 20 10 ppm
